# The structural flexibility of MAD1 facilitates the assembly of the Mitotic Checkpoint Complex

**DOI:** 10.1038/s41467-023-37235-z

**Published:** 2023-03-18

**Authors:** Chu Chen, Valentina Piano, Amal Alex, Simon J. Y. Han, Pim J. Huis in ’t Veld, Babhrubahan Roy, Daniel Fergle, Andrea Musacchio, Ajit P. Joglekar

**Affiliations:** 1 Biophysics, University of Michigan, Ann Arbor, MI 48109 USA; 2 Department of Mechanistic Cell Biology, Max Planck Institute of Molecular Physiology, Dortmund, 44227 Germany; 3 Cell and Developmental Biology, University of Michigan Medical School, Ann Arbor, MI 48109 USA; 4 Centre for Medical Biotechnology, Faculty of Biology, University Duisburg-Essen, Essen, 45141 Germany; 5 Present Address: Department of Molecular Genetics of Ageing, Max Planck Institute for Biology of Ageing, Cologne, 50931 Germany; 6 Present Address: Institute of Human Genetics, University Hospital Cologne, Cologne, 50931 Germany; 7 Present Address: Medical Scientist Training Program, University of Cincinnati College of Medicine, Cincinnati, OH 45267 USA

**Keywords:** Checkpoints, Biocatalysis, Fluorescence imaging, Cell-cycle proteins

## Abstract

The spindle assembly checkpoint (SAC) safeguards the genome during cell division by generating an effector molecule known as the Mitotic Checkpoint Complex (MCC). The MCC comprises two subcomplexes: BUBR1:BUB3 and CDC20:MAD2, and the formation of CDC20:MAD2 is the rate-limiting step during MCC assembly. Recent studies show that the rate of CDC20:MAD2 formation is significantly accelerated by the cooperative binding of CDC20 to the SAC proteins MAD1 and BUB1. However, the molecular basis for this acceleration is not fully understood. Here, we demonstrate that the structural flexibility of MAD1 at a conserved hinge near the C-terminus is essential for catalytic MCC assembly. This MAD1 hinge enables the MAD1:MAD2 complex to assume a folded conformation in vivo. Importantly, truncating the hinge reduces the rate of MCC assembly in vitro and SAC signaling in vivo. Conversely, mutations that preserve hinge flexibility retain SAC signaling, indicating that the structural flexibility of the hinge, rather than a specific amino acid sequence, is important for SAC signaling. We summarize these observations as the ‘knitting model’ that explains how the folded conformation of MAD1:MAD2 promotes CDC20:MAD2 assembly.

## Abstract (web summary)

The formation of the mitotic checkpoint complex requires an intricate spatial coordination amongst the proteins Bub1, Mad1, Cdc20, and Mad2. Chen et al show that a structural flexibility in Mad1 plays an essential role in achieving this coordination.

## Introduction

During mitosis, a eukaryotic cell divides into two genetically identical daughter cells. To achieve this, the duplicated chromosomes in the parent cell must be equally distributed into the daughter cells. The spindle assembly checkpoint (SAC) serves as a surveillance mechanism to ensure that duplicated chromosomes are stably attached to spindle microtubules through an adapter structure named the kinetochore. Kinetochores lacking end-on microtubule attachment activate the SAC to prevent premature anaphase onset and avoid chromosome missegregation. The effector molecule generated upon SAC activation is the Mitotic Checkpoint Complex (MCC). The MCC consists of two subcomplexes: BUBR1:BUB3 and CDC20:MAD2^1,2^. It inhibits the E3 ubiquitin ligase Anaphase-Promoting Complex/Cyclosome (APC/C)^3–5^. APC/C ubiquitinates Cyclin B1, a key mitosis regulator, thereby targeting it for proteasome-mediated degradation^6–8^. Inhibition of the APC/C suppresses the degradation of Cyclin B1, which in turn delays anaphase onset.

The formation of the CDC20:MAD2 complex has been identified as the rate-limiting step in the assembly of the MCC^9,10^. Other checkpoint proteins, including the MAD1:MAD2 complex and the BUB1:BUB3 complex, catalyze this reaction, by recruiting the MCC subunits at kinetochores and facilitating their interaction. CDC20:MAD2 formation also requires the conversion of MAD2 from the “open” conformation (O-MAD2) to the “closed” conformation (C-MAD2)^11–14^. During this conversion, the C-terminal “safety belt” of MAD2 embraces the flexible MAD2-interacting motif (MIM) of CDC20^2,13^. Purified monomeric O-MAD2 spontaneously converts into C-MAD2 at 30 °C in vitro with kinetics that are orders of magnitude slower than expected to support robust CDC20:MAD2 formation during mitosis^15^. In a reconstituted reaction in vitro, MAD1:MAD2 and BUB1:BUB3 were shown to dramatically accelerate the assembly of the CDC20:MAD2 complex, suggesting that they act as the catalysts in the assembly reaction^10,16^. The MAD2 template model^14^ argues that the conformational switch is facilitated by the dimerization between the C-MAD2 bound to MAD1’s MIM in the MAD1:MAD2 complex and a cytosolic O-MAD2 that undergoes the conformational switch to bind CDC20. Furthermore, two recent studies show that the docking of CDC20 on multiple interfaces on MAD1 and BUB1 enables spatiotemporal coupling of the MAD2 conformational switch with its binding to CDC20 thereby overcoming the rate-limiting step and accelerating MCC assembly^16,17^. The exact molecular mechanism of this coupling, however, remains to be elucidated.

In this paper, supported by structural modeling of the human MAD1:MAD2 complex, we hypothesized that efficient CDC20:MAD2 formation may require a folded conformation of the MAD1 C-terminal region, which spans residues 485-718 and includes the MAD1 C-terminal domain (MAD1-CTD)^10,13,17–21^. In agreement with this hypothesis, Fluorescence-Lifetime IMaging (FLIM) suggests that the C-terminal hinge of MAD1 enables the MAD1:MAD2 complex to take a folded conformation in vivo. Importantly, disrupting the structural flexibility of MAD1 by removing the hinge impairs the rate of MCC assembly in vitro and the SAC signaling activity in vivo. Mutating this region while keeping its flexibility maintains the SAC signaling activity, indicating that the structural flexibility of MAD1, rather than the specific amino sequence of the hinge region, is important to the SAC. We propose a “knitting model” that describes how the MAD2 conformational switch is coupled to the formation of CDC20:MAD2, which is key for rapid activation of the SAC in living cells.

## Results

### Structural modeling predicts that the MAD1:MAD2 complex may assume a folded conformation

**Fig. 1:**
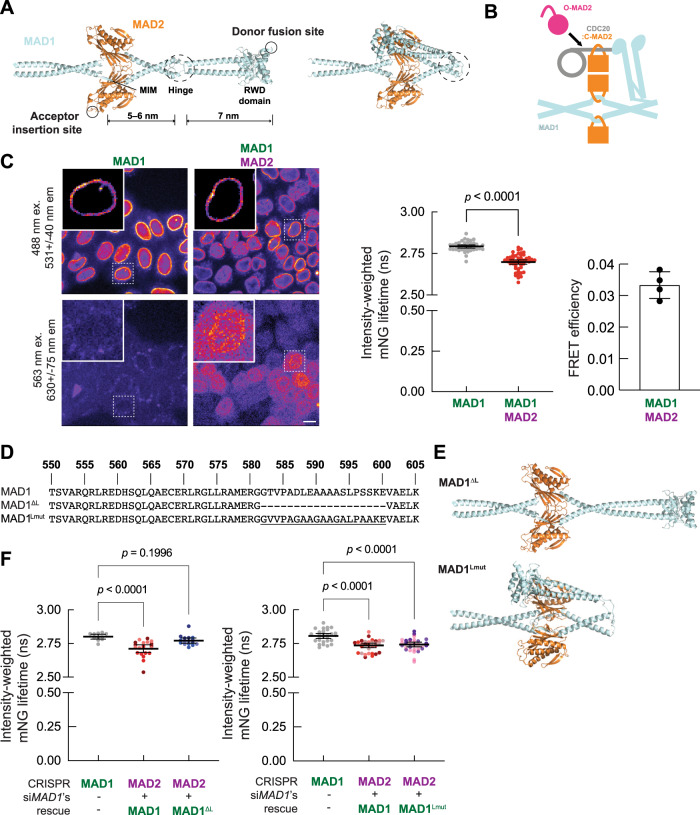
The MAD1:MAD2 complex can assume a folded conformation in vivo enabled by the hinge of MAD1. **A** Representative models of the C-terminal region of the MAD1:MAD2 complex predicted by the ColabFold advanced algorithm^25^ show either extended (left) or folded (right) conformations. These predicted structures agree with published crystal structures, from which labeled length measurements were taken (PDB IDs: 1GO4^13^ and 4DZO^18^). **B** A cartoon demonstrating how the folded conformation of MAD1 may help present the MIM of CDC20 to MAD2. The N-terminal region of CDC20 (including BOX1 and the MIM) —light-gray line; the WD40 fold— light-gray circle^16,21,40^. **C** Left: exemplary images of the genome-edited HeLa-A12 cells expressing both MAD1-mNG and MAD2∨mScarlet-I acquired for FLIM (scale bar ~10 μm). The photon count heatmaps in the MAD1-mNG insets display regions of interest manually thresholded to isolate pixels corresponding to the nuclear envelope. Middle: The excited-state lifetime of MAD1-mNG in the *MAD1*-mNG or the *MAD1*-mNG/*MAD2*∨mScarlet-I genome-edited HeLa-A12 cell lines (*N* = 40 and 52 nuclei, respectively, pooled from four technical repeats). Right: calculated FRET efficiency for MAD1-mNG and MAD2∨mScarlet-I. Two-sided, unpaired *t* tests with Welch’s correction were performed. **D** Partial sequence of the hinge region of human wild-type MAD1, MAD1^ΔL^, and MAD1^Lmut^. **E** A representative model of the C-terminal region of MAD1^ΔL^:MAD2 (top) and MAD1^Lmut^:MAD2 (bottom) predicted by the ColabFold advanced algorithm. **F** Left: the lifetime of MAD1-mNG alone, MAD1-mNG in the presence of MAD2∨mScarlet-I, and MAD1^ΔL^-mNG in the presence of MAD2∨mScarlet-I (*N* = 11, 20, and 16 nuclei respectively pooled from four independent experiments). Right: The average lifetime of MAD1-mNG alone, MAD1-mNG in the presence of MAD2∨mScarlet-I, and MAD1^Lmut^-mNG in the presence of MAD2∨mScarlet-I (*N* = 28, 31, and 32 nuclei, respectively, pooled from three independent experiments). **C**, **F** Each dot represents a single cell. Mean values ± 95% confidence intervals are overlaid. **F** Observations are color-coded by trial number. Source data are provided as a Source Data file.

The MAD1:MAD2 complex is a 2:2 heterotetramer. Prior studies have defined the structures of two nonoverlapping, dimeric segments of the C-terminal region of this heterotetramer: one spanning residues 485–584 of MAD1 complexed with MAD2, and the other, termed as the MAD1-CTD, spanning residues 597–718^13,18^. The SAC kinase MPS1 phosphorylates T716 within the RING finger-containing proteins, WD repeat-containing proteins, and DEAD-like helicases (RWD) domain at the C-terminus of MAD1. Upon phosphorylation, MAD1-CTD binds the BOX1 motif in the N-terminal region of CDC20^16,21^, and this interaction is critical for MCC assembly^10,21,22^. It likely facilitates the coupling of the MAD2 conformational switch with CDC20 binding. However, if we estimate the length of the disordered N-terminus of human CDC20 using the simple 3-D random walk model, the calculated root-mean-square distance from BOX1 (27–34) to MIM (129–133) is less than 4 nm. The worm-like chain model with a persistence length of 0.3–0.7 nm estimates the root-mean-square distance to be 4.5–7.4 nm^23,24^. On the other hand, the combined axial length from the MAD1 MIM to the RWD domain is over 12 nm, according to the crystal structures of the two MAD1:MAD2 segments (Fig. 1A, left panel)^13,18^. Therefore, the flexibility of the CDC20 N-terminus may not be sufficient to position the MIM of CDC20 proximally with respect to MAD2, and additional mechanisms may facilitate the efficient capture of the CDC20 MIM by MAD2.

To gather possible clues, we used AlphaFold2^25,26^ to predict how the structurally known segments of the MAD1 C-terminal region may be arranged. This analysis predicted the existence of folded conformations of MAD1, which are enabled by a flexible hinge spanning residues 582 and 600, in addition to an extended conformation (Fig. 1A, right panel). We reasoned that the folded MAD1 conformation would permit the phosphorylated C-terminal RWD domains of MAD1 to approach the reaction center of the MAD1:MAD2 template complex where O-MAD2 is expected to undergo the conformational switch and bind CDC20 (Fig. 1B). Interestingly, the primary sequence of the hinge region is not conserved from yeast to human (Supplementary Fig. 1A), but an interruption of the coiled-coil with a disordered hinge appears to be very common (Supplementary Fig. 1B). According to AlphaFold2’s predictions, the flexibility of the hinge region enables MAD1 to assume a spectrum of conformations, from fully extended to folded (Fig. 1A)^25,26^.

### Assessment of the in vivo conformation of the MAD1:MAD2 complex using Förster resonance energy transfer (FRET)

To test whether the MAD1:MAD2 complex assumes a folded conformation in vivo, we resorted to distance-sensitive FRET measurements. The folded MAD1 conformation is expected to drastically reduce the distance between the RWD domain and the MIM, and a correctly designed FRET sensor may be able to differentiate such folded conformations from the extended conformation (Fig. 1A). Using CRISPR-Cas9-mediated genome editing, we fused the donor fluorophore mNeonGreen (mNG) to the C-terminal end of MAD1^27^ and inserted the acceptor fluorophore mScarlet-I^28^ in the β5-αC loop of MAD2^29^ (henceforth referred to as MAD2∨mScarlet-I; Fig. 1A and Supplementary Fig. 2A, C). This strategy positions the acceptor fluorophore away from the functional interfaces of MAD2 (the homodimerization interface, the safety belt, and the interface between MAD2 and BUBR1 in the MCC)^2,14,29,30^. The internally tagged Mad2 restored SAC signaling in a budding yeast *Saccharomyces cerevisiae* strain lacking Mad2 (*Δmad2*, Supplementary Fig. 2B), whereas N- or C-terminally tagged Mad2 do not similarly support SAC signaling (our unpublished observations). We confirmed the expression of full-length MAD2∨mScarlet-I in the heterozygous *MAD2*∨mScarlet-I genome-edited HeLa-A12 cell line and verified that the expression level of either BUBR1 or CDC20 was not affected (Supplementary Fig. 2D). Importantly, MAD2∨mScarlet-I partially restored the SAC signaling activity when endogenous MAD2 is knocked down via RNA interference (Supplementary Fig. 2E).

The positioning of the donor and acceptor fluorophores (Fig. 1A) in the extended MAD1 conformation indicates that they will be separated by more than 10 nm, preventing FRET^31^. Conversely, in the folded MAD1 conformation, the donor–acceptor separation will be reduced, enabling FRET (Fig. 1A). Using FLIM^32^, we quantified the excited-state lifetime of MAD1-mNG in the cell line that only expresses MAD1-mNG and the cell line that expresses both MAD1-mNG and MAD2∨mScarlet-I. We conducted these measurements on MAD1:MAD2 complexes localized to the nuclear pore complex (NPC) in interphase cells^33^ (Fig. 1C). We expected that the relatively sparse MAD1:MAD2 localization over the entire nuclear membrane would minimize inter-complex FRET, which likely exists between MAD1:MAD2 complexes localized to the corona of a signaling kinetochore in the prometaphase^31^. We found that the excited-state lifetime of MAD1-mNG in the presence of MAD2∨mScarlet-I was modestly lower than the lifetime of MAD1-mNG alone (2.69 ± 0.05 vs. 2.79 ± 0.03 ns, respectively, Fig. 1C, middle; also see Supplementary Fig. 3). These average lifetime values indicate that the efficiency of FRET between MAD1-mNG and MAD2∨mScarlet is 3.6% (Fig. 1C, right). The modest FRET can be attributed, in part, to the presence of endogenous, unlabeled MAD1 and MAD2 in our heterozygous cell line.

Because of the modest FRET efficiency observed, we performed several control experiments to establish the efficacy of our FLIM acquisition and analysis protocol. We transiently expressed fusions of either mNG, mScarlet-I, or a tandem mNG-mScarlet-I tag to the C-terminus of NUP50, which is a component of the nuclear basket^34^ (Supplementary Fig. 3A). The fluorescent protein fused to NUP50 should experience a similar micro-environment as MAD1-mNG. Consistent with this, the NUP50*-*mNG fluorescence lifetime was indistinguishable from the MAD1*-*mNG lifetime. As expected, the lifetime of the mNG in NUP50*-*mNG-mScarlet-I was significantly lower (2.78 ± 0.03 and 2.16 ± 0.14 ns, respectively, Supplementary Fig. 3D). These values indicate a FRET efficiency for NUP50*-*mNG-mScarlet-I of 22%, which is comparable to the reported FRET efficiency values spanning 30–40% for a cytoplasmic EGFP-mCherry tandem FRET pair^35^. Importantly, the lifetime of MAD1-mNG in the presence of NUP50*-*mScarlet-I was only slightly lower than the lifetime of MAD1-mNG alone (2.77 ± 0.02 vs. 2.79 ± 0.03, respectively), indicating negligible FRET (Supplementary Fig. 3D). These data validate our FLIM acquisition and analysis protocols.

### Structural flexibility of the MAD1 hinge is required for FRET between MAD1-mNG and MAD2∨mScarlet-I

To reinforce these observations and test whether the MAD1 hinge is needed to enable FRET between MAD1*-*mNG and MAD2∨mScarlet-I, we designed two MAD1 mutants: MAD1^ΔL^ and MAD1^Lmut^. In MAD1^ΔL^, we deleted the hinge (a.a. residues 582–600, Fig. 1D) while preserving the heptad repeat periodicity of the upstream and downstream coiled-coils predicted by MARCOIL and DeepCoil2^36–38^. AlphaFold2 predicts an uninterrupted and fully extended coiled-coil for MAD1^ΔL^ (Fig. 1E, top). In MAD1^Lmut^, we replaced the original hinge with an alanine-rich, non-phosphorylatable peptide of the same length. AlphaFold2 predicts folded conformations for MAD1^Lmut^ similar to that of wild-type MAD1 (Fig. 1E, bottom).

We used the knockdown/knock-in strategy to replace the endogenous, unlabeled MAD1 with MAD1(WT/ΔL/Lmut)-mNG (see Methods). We found a statistically significant increase in the lifetime of MAD1^ΔL^-mNG compared to MAD1-mNG (2.77 ± 0.04 and 2.71 ± 0.06, respectively, scatterplot on the left in Fig. 1F). Furthermore, the excited-state lifetime of MAD1^ΔL^-mNG in the presence of MAD2∨mScarlet-I was statistically indistinguishable from the excited-state lifetime of MAD1-mNG alone (Fig. 1F). In contrast, in the presence of MAD2∨mScarlet the excited-state lifetimes of MAD1^Lmut^-mNG and MAD1-mNG were the same (2.74 ± 0.05 and 2.74 ± 0.04, respectively, scatterplot on the right in Fig. 1F). These results support our conclusion that the structural flexibility of the C-terminus of MAD1 enabled by the hinge facilitates folding of the MAD1 C-terminal region in vivo. Finally, the FRET between MAD1-mNG and MAD2∨mScarlet-I persists even when even when *CDC20* is knocked down by RNAi (Supplementary Fig. 3E), suggesting that the folded conformation is an intrinsic property of the MAD1:MAD2 complex.

### MAD1’s hinge is important to the rate of MCC assembly in vitro

**Fig. 2:**
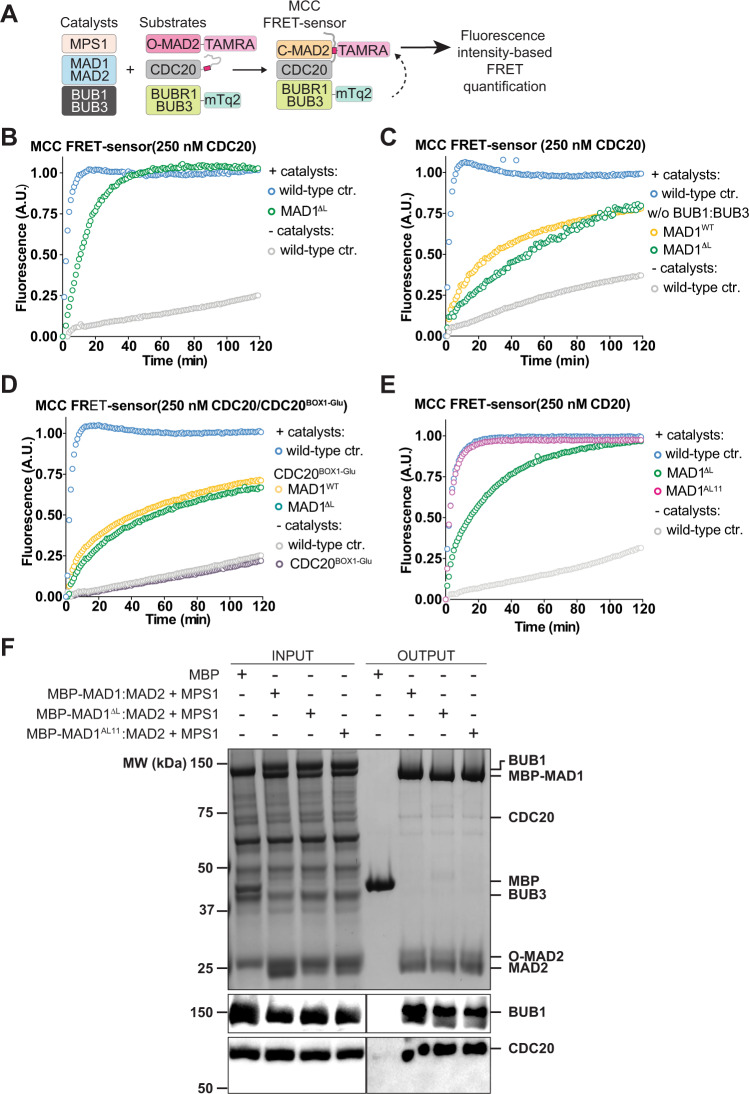
The rate of MCC assembly is lower in the presence of MAD1ΔL than in the presence of wild-type MAD1 in vitro. **A** The biochemical scheme of the FRET sensor which quantifies the assembly rate of the MCC^10,16^. In this sensor, mTurquoise2-BUBR1 (mTq2-BUBR1) and MAD2-TAMRA serve as the donor and the acceptor, respectively. Upon MCC assembly, mTurquoise2 and TAMRA will be closely positioned, allowing FRET. FRET was quantified as the sensitized TAMRA fluorescence emission (see “Methods”). **B** The addition of MBP-MAD1^ΔL^:MAD2 (green) causes a moderate decrease in the rate of MCC assembly compared to the wild-type (blue). **C**, **D** MBP-MAD1:MAD2 (yellow) and MBP-MAD1^ΔL^:MAD2 (green) have similar MCC assembly rates (**C**) in the absence of BUB1:BUB3 or (**D**) when CDC20^BOX1-Glu^ is used in the reaction instead of wild-type CDC20. **E** MBP-MAD1^AL11^:MAD2 (magenta) can promote MCC assembly in vitro similarly to wild-type MBP-MAD1:MAD2 (blue). **B**–**E** Curves report single measurements and are representative of at least three independent technical replicates. The *y* axis represents the normalized emission intensity of the acceptor. **F** MBP or MBP-MAD1 (wild-type or mutant):MAD2 is immobilized on amylose beads and serves as baits to pull down prey including O-MAD2 (a V193N mutant that stabilizes MAD2 in the open conformation^29^), MPS1-phosphorylated BUB1:BUB3, and CDC20. From top to bottom: a Coomassie-stained SDS-PAGE gel, an immunoblot detecting BUB1, and an immunoblot detecting CDC20. Source data are provided as a Source Data file.

To test the role of the structural flexibility of MAD1 in the assembly of the MCC, we purified recombinant MAD1:MAD2 and MAD1^ΔL^:MAD2. Importantly, these complexes appeared stable and properly folded (Supplementary Fig. 4A). We compared their MCC assembly activity in vitro using the previously established MCC FRET-sensor-based assays (Fig. 2A)^10,16^. In this assay, MCC assembly is monitored by quantifying the FRET intensity signal arising from the close proximity between mTurquoise2-BUBR1 (the donor fluorophore) and MAD2-TAMRA (the acceptor fluorophore, see Fig. 2A).

Deletion of MAD1’s hinge caused a moderate but reproducible decrease in the rate of MCC assembly compared to the wild-type (Fig. 2B), indicating that the hinge is important to maximize the rate of MCC assembly in vitro. The rate difference between MAD1:MAD2 and MAD1^ΔL^:MAD2 relied on the presence of BUB1:BUB3 (Fig. 2C). More specifically, the rate difference required a functionally intact BUB1:BUB3 complex to interact with MAD1:MAD2, because the BUB1^ΔCM1^ mutant that prevents this interaction erased the difference (Supplementary Fig. 4B).

Manifestation of a rate difference between MAD1:MAD2 and MAD1^ΔL^:MAD2 also relied on the interaction of CDC20 with MAD1, as it was abolished by mutation of the BOX1 motif of CDC20 (Fig. 2D). Collectively, these observations suggest that flexibility enabled by the hinge region allows MAD1:MAD2 to interact more productively with BUB1 and CDC20 during the catalytic conversion that promotes MCC assembly. In a solid phase binding assay with immobilized MAD1:MAD2, we found that the binding of O-MAD2, BUB1, and CDC20 was not overtly affected by the hinge-deletion mutation (Fig. 2F). We conclude that the role of the structural flexibility of MAD1 in the rate of MCC assembly in vitro is critical to the appropriate spatial association of BUB1, CDC20, and MAD1:MAD2^10,16,17^ (see “Discussion”).

### The C-terminal hinge of MAD1 is important to the SAC signaling activity in vivo

**Fig. 3:**
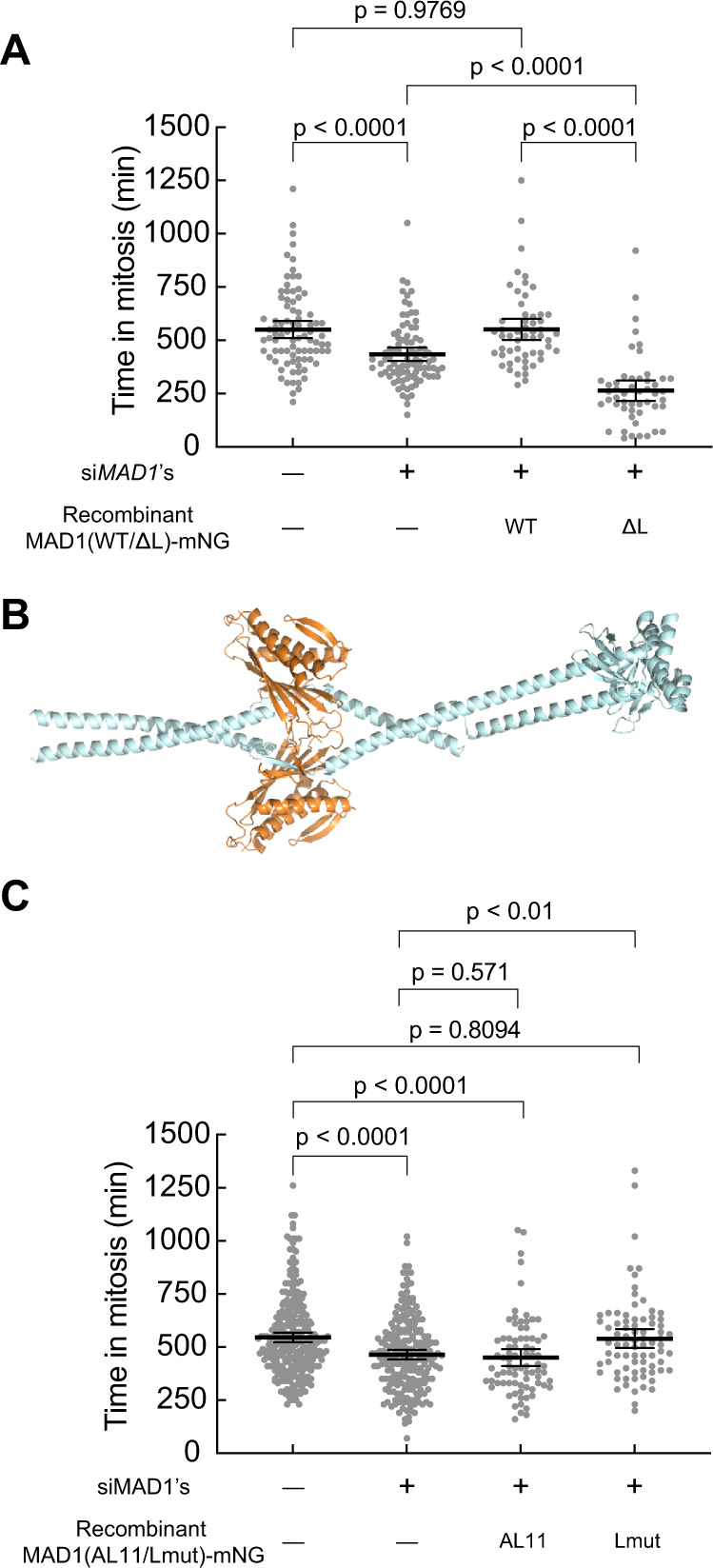
The structural flexibility provided by the hinge of MAD1 is critical to the SAC signaling activity in vivo. **A** The first two columns on the left used the *MAD1*-mNG genome-edited HeLa-A12 cell line which served as a reference for the endogenous level of MAD1 (see “Methods”). In situ tagging of MAD1 did not affect the 3’-UTR which si*MAD1*’s target. The two columns on the right used HeLa-A12 cells treated with si*MAD1*’s and induced to express exogenous MAD1-mNG or MAD1^ΔL^-mNG. Each dot represents a cell (*N* = 86, 89, 55, and 50, respectively). **B** In the predicted structure of the core region of the MAD1:MAD1^ΔL^ heterodimer (in complex with MAD2, using the ColabFold advanced algorithm), the hinge of the wild-type copy introduces a bulge but the overall conformation is extended due to the stiffness of the now fused α-helix of MAD1^ΔL^. **C** As in (**A**), the first two columns on the left used the *MAD1*-mNG genome-edited HeLa-A12 cell line which served as a reference for the endogenous level of MAD1. The two columns on the right used HeLa-A12 cell lines treated with si*MAD1*’s and induced to express exogenous MAD1^AL11^-mNG or MAD1^Lmut^-mNG. Each dot represents a cell (*N* = 265, 219, 80, and 78, respectively). **A**, **C** Results were pooled from at least two technical repeats. The mean value ± the 95% confidence interval of each group is overlaid. Unpaired two-sided *t* tests with Welch’s correction are performed in Prism. Source data are provided as a Source Data file.

Next, we sought to determine whether the C-terminal hinge of MAD1 is important for SAC signaling in vivo. Cells with less than 10% of the physiological level of MAD1 generally retained a robust checkpoint response in 100 nM nocodazole that could not be weakened by increasing the dosage of si*MAD1*’s (Supplementary Fig. 5A, B). Nonetheless, SAC signaling activity was crippled, as MAD1-depleted cell sexited mitosis at least 2 h earlier than the untreated control cells (Fig. 3A). In this context, however, expression of MAD1^ΔL^-mNG resulted in a dominant-negative effect that considerably shortened the mitotic arrest. For comparison, wild-type MAD1-mNG restored the SAC signaling activity to levels observed in the negative control (Fig. 3A). We reasoned that the dominant-negative effects of MAD1^ΔL^-mNG reflect its dimerization with the residual endogenous MAD1 and consequent restriction of its structural flexibility. Indeed, structural predictions of MAD1:MAD1^ΔL^ suggested that the hinge region of wild-type MAD1 cannot adopt the folded conformation when facing the stiff continuous α-helix of the MAD1^ΔL^ counterpart (Fig. 3B). To test this experimentally, we pulled down doxycycline-induced MAD1(wild-type/ΔL)-mNG from lysates of HeLa-A12 cells in which endogenous MAD1 was not knocked down. We found that endogenous MAD1 was pulled down both by MAD1-mNG and by MAD1^ΔL^-mNG, but not by mNG alone (Supplementary Fig. 6). We further confirmed that MAD1^ΔL^-mNG did not cause defects in the localization of the MAD1^ΔL^:MAD2 complex (Supplementary Fig. 5C) or the expression of BUBR1, CDC20, or BUB3 (Supplementary Fig. 5B). Therefore, although the results of our knockdown-rescue experiments were hindered by the incomplete knockdown of the endogenous MAD1, all evidence combined suggested that the hinge of MAD1 is critical for the SAC.

### MAD1^Lmut^ fully supports the SAC signaling activity in vivo

The observation that the hinge encompassing residues 582–600 of MAD1 is important for SAC signaling in vivo may have alternative explanations. For instance, it is known that S598 can be phosphorylated by MPS1 in vitro^21^. The hinge of MAD1 may also be required for unknown protein–protein interactions important to the SAC. To distinguish among these possibilities, we reasoned that replacing the hinge with an equally flexible region of a diverged sequence should prevent sequence-specific physical interactions with putative binding partners while preserving MAD1’s ability to adopt the folded conformation. Therefore, we tested MAD1^Lmut^ and an additional MAD1 mutant named MAD1^AL11^, wherein the 19 residue span of the MAD1 hinge with a previously characterized flexible linker^39^ (sequence shown in Supplementary Fig. 7A). MAD1^AL11^ is also predicted to have a coiled-coil propensity profile similar to that of MAD1 (Supplementary Fig. 7A). However, unlike MAD^Lmut^, the hinge of MAD1^AL11^ does not contain the two proline residues, which are commonly found within the hinge of MAD1 across phylogeny (Supplementary Fig. 2B).

We observed that MAD1^AL11^:MAD2 had the same MCC assembly activity as the wild-type complex in our in vitro assay (Fig. 2E). We were unable to purify recombinant MAD1^Lmut^:MAD2, possibly because of instability during protein purification introduced by the mutation. Both mutants were correctly expressed in HeLa cells (Supplementary Fig. 7B). Furthermore, in cells treated with si*MAD1* and expressing MAD1^AL11^-mNG, SAC signaling was weaker than in cells expressing wild-type MAD1, whereas MAD1^Lmut^-mNG fully restored the SAC signaling activity (Fig. 3C). We conclude that MAD1^Lmut^ with its artificial hinge is completely checkpoint proficient, contrary to MAD1^ΔL^. Conversely, the more flexible loop in MAD1^AL11^ does not restore full activity in vivo. Impaired activity of MAD^AL11^ is not due to defects in its localization to unattached kinetochores (Supplementary Fig. 7C). The main difference between the checkpoint proficient MAD1^Lmut^ and the checkpoint defective MAD1^AL11^ is that the former contains two proline residues that are absent in the latter. We surmise therefore that these proline residues may be important for the structural conversion we have identified. Even if MAD1^AL11^ appeared to be as active as the wild-type MAD1 in the in vitro MCC generation assay (see Fig. 2E), we attribute the discrepancy with the in vivo result to the reduced sensitivity of the assay in vitro relative to the more stringent checkpoint signaling in vivo. Indeed, we have observed a similar outcome with a small subset of additional mutations known to cause checkpoint defects in vivo^16^. Collectively, our observations suggest that the primary function of the hinge is providing structural flexibility rather than mediating unspecified protein–protein interactions.

## Discussion

**Fig. 4:**
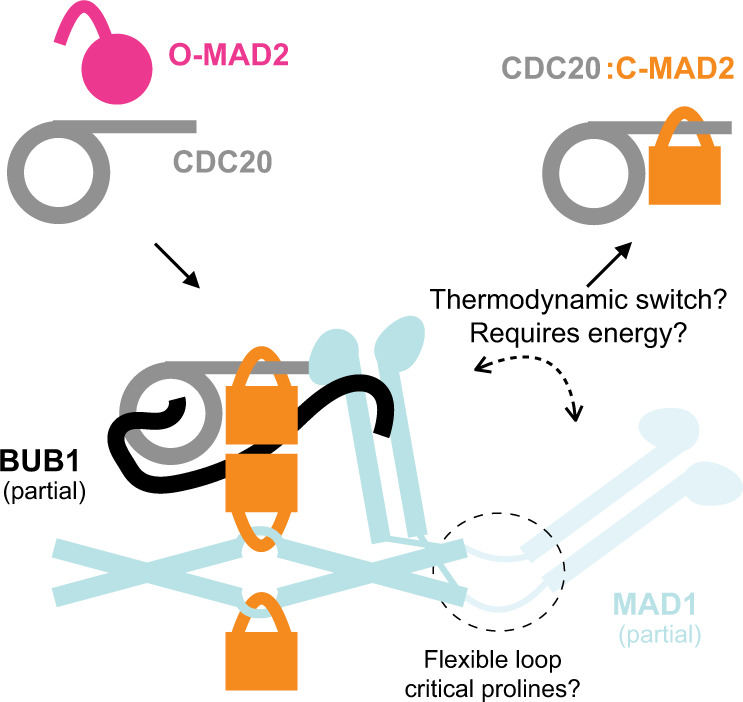
A cartoon of the knitting model. The structural flexibility of MAD1 facilitates a spatiotemporal coupling of the MAD2 conformational switch and the assembly of CDC20:MAD2. The two solid black arrows indicate the formation and release of CDC20:MAD2, respectively. According to Fig. 2C and Supplementary Fig. 4B, the difference in the MCC assembly rate (comparing MAD1 with MAD1^ΔL^) relies on the interaction between MAD1 and BUB1. Therefore, this cartoon of our model also incorporates BUB1 and highlights the following protein–protein interactions involving BUB1: (1) T461-phosphorylated BUB1 CM1 interacts with MAD1’s consensus RLK motif located within the coiled-coil leading up to the RWD domain^21,56^; (2) the C-terminus of BUB1 CM1 contacts the RWD domain of the opposite MAD1^56^; (3) BUB1 interacts with CDC20 through multiple motifs cooperatively, including the ABBA motif (527–532, which binds between blades 2 and 3 of CDC20’s seven-bladed WD40 fold) and the consensus KEN box (C-terminal to the ABBA motif, which likely binds to the center of CDC20’s WD40)^16,57,58^.

Here, we identified a previously unrecognized molecular mechanism that helps overcome the kinetic barrier associated with the binding of MAD2 and CDC20. A folded conformation of MAD1 positions the MIM of CDC20 and MAD2 closely, facilitating the assembly of the CDC20:MAD2 complex. In a parallel study^40^, Fischer and colleagues demonstrate that the CM1 of human BUB1 and the α1 helix of CDC20, which precedes BOX1, interact in a tripartite 1:1:2 complex with the RLK motif of MAD1. Thus, collectively, CDC20 establishes multiple interfaces with the catalysts BUB1 and MAD1:MAD2, and these interactions likely position the CDC20 MIM for its efficient capture by MAD2. Switching back to an extended conformation may break the avidity, thereby releasing assembled CDC20:MAD2 into the cytosol. We use the knitting analogy to describe this model (Fig. 4), as the two MAD1 functional regions connected by the hinge switch their relative positioning and work coordinately like two knitting needles to entangle CDC20 and MAD2.

In the aforementioned parallel study^40^, the purified MAD1:MAD2 complex was shown to exhibit a folded conformation in vitro. Here, we showed that the MAD1:MAD2 complex assumes such a folded conformation also in vivo. Our data indicate that the structural flexibility is enabled by a flexible hinge in the C-terminus of MAD1, whose secondary structure—rather than the primary sequence—is conserved. This hinge is important for MCC assembly in vitro and SAC signaling in vivo, and we provide evidence that it can be replaced with similarly flexible but different sequences, implying that the hinge is unlikely to mediate hitherto unknown physical interactions with other proteins. Thus, collectively, the structural flexibility of MAD1 appears to be important to the SAC signaling activity.

Whether MAD1 switches between an extended conformation and the folded conformation at a physiologically meaningful rate in vivo, and whether this switching cycle correlates with the formation of a CDC20:MAD2 complex is currently unclear. The distribution of conformations of the two proline residues (P585 and P596) in the hinge may be under active, energy-consuming regulation in the cell, but assessing this will require further analyses. We note that no MAD1-interacting protein with peptidylprolyl cis-trans isomerase activity has been identified in the PrePPI database as of March 2022^41,42^. It remains unknown whether the proline residues simply serve to break the coiled-coil or play a more complex role in promoting the folding of MAD1.

Our in vitro reconstitution data suggest that the critical role of the flexibility of MAD1 is strictly coupled with BUB1. In the absence of BUB1 in the reactions, the assembly rates of CDC20:MAD2 were the same for both MAD1 and MAD1^ΔL^. However, assembly of MCC continues during interphase and prophase^43^. There has been no report on BUB1’s localization at the NPC where the MAD1:MAD2 complex is predominantly localized during the interphase and prophase. Therefore, either the flexibility of MAD1 alone scaffolds CDC20:MAD2 coupling at the NPC or there may be a nucleoporin that functions similarly to BUB1. Future studies should examine how the MAD1:MAD2 complex may catalyze the formation of the CDC20:MAD2 complex at the NPC during the interphase and prophase.

## Methods

Wide-field, *z*-stack fluorescence imaging for the quantification of the localization of MAD1-mNG, MAD1^ΔL^-mNG, and MAD2∨mScarlet-I at signaling kinetochores was the same as described previously^44^. AlphaFold2 structural predictions were conducted using the ColabFold advanced algorithm^25^. All ColabFold parameters were set to their default values except for “max recycles” (which was set to 6) and “tol” (which was set to 0.1).

### Theoretical end-to-end root-mean-square distance (RMSD) of a flexible unstructured peptide

First, we model a flexible peptide with *n* amino acid residues as a 3-D random walk (without considering steric hindrance and restrictions imposed by the Ramachandran plot). We denote the displacement of residue number *i* + 1 relative to residue number *i* as a random vector **r**_*i*_, *i* = 1, 2,…, *n* − 1. The end-to-end displacement, **D**, can be expressed as

1 $${{{{{\bf{D}}}}}}=\sum _ {i=1}^{n-1}{{{{{{\bf{r}}}}}}}_{i}$$

The RMSD is therefore

2 $$\sqrt{\left\langle {\left|{{{{{\bf{D}}}}}}\right|}^{2}\right\rangle }=\sqrt{{\sum }_{i=1}^{n-1}\left\langle {\left|{{{{{{\bf{r}}}}}}}_{i}\right|}^{2}\right\rangle+{\sum }_{i\ne j}\left\langle {{{{{{\bf{r}}}}}}}_{i}\cdot {{{{{{\bf{r}}}}}}}_{j}\right\rangle }$$

For a 3-D random walk, the random vectors representing each step are independent of each other. Therefore, ∀*i* ≠ *j*,

3 $$\left\langle {{{{{{\bf{r}}}}}}}_{i}\cdot {{{{{{\bf{r}}}}}}}_{j}\right\rangle=0$$

Suppose that the contour length of each amino acid residue is universal (|**r**_*i*_ | = *r*, *i* = 1, 2,…, *n* − 1; we take *r* = 0.37 nm here^45^), we have

4 $$\sqrt{\left\langle {\left|{{{{{\bf{D}}}}}}\right|}^{2}\right\rangle }=r\sqrt{n-1}=\frac{L}{\sqrt{n-1}}$$

wherein *L* = (*n* − 1)*r* is the contour length of the peptide.

Next, we model the same peptide using a worm-like chain model^23,24^. This model considers the peptide as a continuous worm-like chain rather than a discrete, step-by-step walk. The end-to-end RMSD is

5 $$\sqrt{\left\langle {\left|{{{{{\bf{D}}}}}}\right|}^{2}\right\rangle }=\sqrt{2{pL}\left[1-\frac{p}{L}(1-{e})^{-\frac{L}{p}}\right]}$$

wherein *p* is the persistence length (we take *p* = 0.3–0.7 nm here^23,24^), a metric for the stiffness of the chain.

### Purification of recombinant proteins

Wild-type or mutant constructs of MAD1:MAD2, MAD2, MPS1, BUB1:BUB3, CDC20, and BUBR1:BUB3 are of human origin. The constructs of MBP-MAD1^ΔL^:MAD2 and MBP-MAD1^AL11^:MAD2 are cloned via site-directed mutagenesis from the MBP-MAD1:MAD2 wild-type construct described previously^10,16^. All recombinant proteins used in this study have been expressed and purified according to the protocols described previously^10,16^.

### Low-angle metal shadowing and electron microscopy

MBP-MAD1:MAD2 or MBP-MAD1^ΔL^:MAD2 was diluted 1: 1 with a spraying buffer (200 mM ammonium acetate and 60% glycerol) to a final concentration of 0.5–1.0 μM and air-sprayed onto freshly cleaved mica pieces (V1 quality, Plano GmbH). Specimens were mounted and dried in a MED020 high-vacuum metal coater (Bal-tec). A 1-nm platinum layer and a 7-nm carbon support layer were subsequently evaporated onto the rotating specimen at angles of 6–7° and 45°, respectively. Pt/C replicas were released from the mica on water, captured by freshly glow-discharged 400-mesh Pd/Cu grids (Plano GmbH), and visualized using a LaB_6_-equipped JEM-1400 transmission electron microscope (JEOL) operated at 120 kV. Images were recorded at a nominal magnification of 60,000× on a 4k × 4k CCD camera F416 (TVIPS).

### FRET assay with the MCC FRET sensor

The MCC FRET sensor has been described previously^10,16^. The catalysts preparation consisted of 2 μM MBP-MAD1(wild-type or mutant):MAD2 and 2 μM BUB1 (wild-type or mutant):BUB3, which were separately incubated with 500 nM MPS1 in the assay buffer [10 mM HEPES (pH 7.5), 150 mM NaCl, 2.5% glycerol, and 10 mM β-mercaptoenthanol] supplemented with 1 mM ATP and 10 mM MgCl_2_ for 16 h at 4 °C. All assays were performed using a 100 nM final concentration of all proteins, except for CDC20, which was added at 250 nM. The fluorophores MAD2-TAMRA and mTurquoise2-BUBR1(1-571):BUB3 were added before measurements started. All measurements were performed on a CLARIOstar plate reader (BMG Labtech), using UV-Star 96-well plates (Greiner). The reactions had a final volume of 100 μL in the assay buffer. The excitation light and emitted fluorescence were filtered by a 430–10 nm excitation filter, an LP 504 nm dichroic mirror, and a 590–20 nm emission filter. The plate reader read at a 60-s interval for 120 min (6 mm focal height, 200 flashes, gain 1200) and mix the reactions for 5 s at 500 revolutions per minute after each measurement.

### Flow cytometry

The complete genotype of the *mad2Δ* S. cerevisiae strain (AJY4951) is *leu2Δ−1, trp1Δ63, ura3-52, his3Δ200, lys2-8Δ1, mad2Δ::TRP1*. The complete genotype of the Mad2∨GFP-expressing *S. cerevisiae* strain (AJY5041, constructed for this study) is *leu2Δ0, met15Δ0, ura3Δ0, mad2Δ::KAN, Mad2101::GFP (HIS3)*.

Yeast strains were grown to mid-log phase, and then 15 μg/mL nocodazole was added to the media. Sample aliquots containing ∼2 × 10^6^ cells were collected 0, 1, 2, 3, and 4 h after the addition of nocodazole. Samples were fixed by 70% ethanol and then stored at 4 °C overnight. On day 2, samples were washed with the RNase buffer [10 mM Tris (pH 8.0), 15 mM NaCl] and treated with 170 ng/mL bovine pancreatic RNase (Millipore Sigma) at 37 °C for one day in the RNase buffer. On day 3, samples were washed again, resuspended in PBS, and stored at 4 °C. The samples were treated with 5 mg/mL propidium iodide (Millipore Sigma) for 2 h at room temperature and subject to flow cytometry on an LSRFortessa Cell Analyzer (BD Biosciences). Approximately 10,000 cells were analyzed from each sample.

Data were analyzed using FlowJo (BD). Cells were first gated based on the area of the forward scatter and side scatter peak, followed by the area of the fluorescence peak (610 nm). Exemplary plots depicting the gating are included in the Source Data file.

### Generating the *MAD2*∨mScarlet-I genome-edited HeLa-A12 cell line

The gRNA used in the integration of the coding sequence of MAD2∨mScarlet-I (intron-free, stop codon-containing, and si*MAD2*-resistant by the introduction of silent mutations) and the polyadenylation signal of rabbit β-globin after the first exon of the endogenous *MAD2* gene was 5’-UCGCGCAGGCCAAUAUAUCG-3’. Synthesis of the sgRNA and assembly of the *Sp*Cas9-sgRNA RNP complex were described in ref. ^27^. HeLa-A12 or the heterozygous *MAD1*-mNG genome-edited HeLa-A12 cell line^27^ was co-transfected with the RNP complex and the linearized homology-directed repair template plasmid (pCC35), sorted by fluorescence-activated cell sorting, and validated as described in ref. ^27^. A successfully edited *MAD2*∨mScarlet-I allele encodes an internally tagged MAD2 protein, wherein MAD2 and the mScarlet-I tag are separated by short flexible linkers (AGSGSGGAS between S114 of MAD2 and the N-terminus of mScarlet-I; GTGAGSA between the C-terminus of mScarlet-I and A115 of MAD2).

### RNA interference

The two siRNAs targeting the 3’-UTR of *MAD1* (si*MAD1*’s)^46^ were applied to unsynchronized cells at a concentration of 40 nM each for 2 days before imaging or cell-harvesting unless specified otherwise. The sense-strand sequence of si*CDC20* was 5’-GGAGCUCAUCUCAGGCCAU-3’^47^, which was applied at a concentration of 40 nM for 2 days before FLIM or cell-harvesting. The sense-strand sequence of si*MAD2* was 5’-GGAAGAGUCGGGACCACAGUU-3’^48^, which was applied at a concentration of 40 nM for 1 day before imaging or cell-harvesting. Desalted double-stranded siRNAs modified by double-deoxythymidine overhangs at 3’-ends of both strands were synthesized by Sigma. AllStars Negative Control siRNA (QIAGEN) is used as the control siRNA (siCtrl) and applied at the same dosage and time as the corresponding experimental group(s). All siRNAs were transfected into the cells via Lipofectamine RNAiMAX (Invitrogen).

### Fluorescence lifetime imaging microscopy (FLIM)

All FLIM data were collected on an ISS Alba v5 Laser Scanning Microscope, connected to an Olympus IX81 inverted microscope equipped with a UPLSAPO60XW objective. A Fianium WL-SC-400-8 laser with an acousto-optic tunable filter was used to generate excitation pulses at a wavelength of 488 nm and a frequency of about 20 MHz. The excitation light was further filtered by a ZT405/488/561/640rpc (Chroma Technology) quadband dichroic mirror. The emission light of the green channel was redirected by a 562 longpass dichroic mirror (FF562-Di03, Semrock), filtered by an FF01-531/40-25 filter (Semrock), and finally detected by an SPCM-AQRH-15 avalanche photodiode (Excelitas Technologies). The time-correlated single photon counting module to register detected photon events to excitation pulses was SPC-830 (Becker & Hickl). Data acquisition was facilitated by VistaVision (ISS).

A multi-component exponential fit is intrinsically flexible^49^. The fluorescence decay of mNeonGreen alone is multi-exponential^50^. Furthermore, the FRET efficiency between MAD1-mNG and MAD2∨mScarlet-I may be variable depending on the conformation of the MAD1:MAD2 complex as well as the presence of possible unlabeled MAD1 and MAD2 molecules in our heterozygous genome-edited cell lines. With these complications in mind, we used a two-component exponential decay model to fit the FLIM data:

6 $$I\left(t\right)=C\left[\alpha {e}^{-\frac{t}{{\tau }_{1}}}+\left(1-\alpha \right){e}^{-\frac{t}{{\tau }_{2}}}\right] \circledast {{{{{\rm{IRF}}}}}}(t)+D$$

In the equation above, *D* is the background signal offset. *τ*_1_ and *τ*_2_ are the lifetimes of the two exponential components. IRF(*t*) is the instrument response function. The IRF was determined by determining the photon arrival histogram for the donor channel using a 500 nM Rose Bengal solution in 5.6 M potassium iodide illuminated with the excitation laser. IRF drift^51,52^ was corrected by translating the IRF along the time axis by up to 2 ps to eliminate exponential components with unrealistic short lifetimes. The MATLAB nonlinear optimization function “fmincon” was used to find the best parameter set that fit the FLIM data. For more details, please refer to the data analysis toolkit available at https://github.com/CreLox/FluorescenceLifetime.

To demonstrate how fluorescence-lifetime measurements can quantify the FRET efficiency, consider a large number of donor fluorophore molecules with a lifetime of *τ*_0_. In the absence of acceptor fluorophores, the exponential decay *D*_0_ of donor fluorescence after pulsed excitation at time zero is

7 $${D}_{0}\left(t\right)=C{e}^{-\frac{t}{{\tau }_{0}}}$$

The total donor fluorescence intensity is

8 $${S}_{0}={\int }_{0}^{+{{\infty }}}{D}_{0}\left(t\right){dt}=C{\tau }_{0}$$

wherein *C* is a constant determined by the total number and properties of fluorophores, as well as the imaging setup. Without altering any of these conditions, in the presence of acceptor fluorophores and FRET, the possibility that an excited fluorophore stays excited (has not relaxed to the ground state either through the fluorescence-emitting route or the FRET-quenching route) at time *t* is

9 $${P=e}^{-\left(\frac{1}{{\tau }_{0}}+\frac{1}{\tau {\prime} }\right)t}$$

wherein *τ'* is the time constant of FRET (although an excited fluorophore can only relax through one route, the two stochastic processes—fluorescence-emitting and FRET-quenching—are independent). Therefore, in the presence of acceptor fluorophores and FRET, the new decay dynamics of the donor fluorescence are

10 $${D\left(t\right)={Ce}}^{-\left(\frac{1}{{\tau }_{0}}+\frac{1}{\tau {\prime} }\right)t}=C{e}^{-\frac{{\tau }_{0}+\tau {\prime} }{{\tau }_{0}\tau {\prime} }t}$$

The effective lifetime of the donor fluorophore (which can be measured through FLIM) becomes

11 $$\tau=\frac{{\tau }_{0}\tau {\prime} }{{\tau }_{0}+\tau {\prime} }$$

and the total donor fluorescence intensity becomes *S* = *Cτ*. Therefore, the FRET efficiency

12 $$\frac{{S}_{0}-S}{{S}_{0}}=\frac{{\tau }_{0}-\tau }{{\tau }_{0}}$$

Because the fluorescence lifetime in the absence of quenching is an intrinsic property of a mature fluorescent protein under a certain temperature^53^, the equation above greatly simplifies experiments to measure the FRET efficiency.

### Time-lapse live-cell imaging in knockdown-rescue mitotic duration assays

Time-lapse live-cell imaging was performed on an ImageXpress Nano Automated Imaging System (Molecular Devices). A SOLA Light Engine (Lumencor) served as the excitation light source. Cells were plated on 24-well cell imaging plates (with black walls and glass bottom, Eppendorf) and treated with siRNAs and 100 nM nocodazole accordingly. Humidified 5% CO_2_ was supplied to the environment chamber maintained at 37 °C.

Using Cre-*lox* recombination-mediated cassette exchange^44,54,55^, we integrated the Tet-On expression cassette of either MAD1-mNG, MAD1^ΔL^-mNG, MAD1^AL11^-mNG, or MAD1^Lmut^-mNG into the HeLa-A12 cell line. According to a previous study^20^, the level of MAD1 and MAD2 must be balanced for a robust SAC. To make sure that the expression of exogenous, si*MAD1*-resistant MAD1(wild-type/mutant)-mNG in si*MAD1*-treated HeLa-A12 cells is close to the physiological level of endogenous MAD1 for all analyzed cells, we always imaged the heterozygous *MAD1*-mNG genome-edited HeLa-A12 cell line^27^ as the control in all our knockdown-rescue mitotic duration assays. Therefore, only cells with green fluorescence intensity (after correcting for the background and shading) close to two times the green fluorescence intensity in the heterozygous *MAD1*-mNG genome-edited HeLa-A12 cell line were analyzed in our knockdown-rescue mitotic duration assays.

### Pulldown using amylose beads

BUB1:BUB3, CDC20, O-MAD2(V193N), and MBP-MAD1(wild-type or mutant):MAD2 were diluted using a binding buffer [20 mM HEPES (pH 7.5), 150 mM NaCl, 5% (by volume) glycerol] in a total volume of 50 μL. Unless specified otherwise, MBP-MAD1 (wild-type or mutant):MAD2 and BUB1:BUB3 were diluted at 20 μM and pre-phosphorylated at 4 °C for 16 h by MPS1 (1 μM). The final concentration of MBP or MBP-MAD1 (wild-type or mutant):MAD2 was 4 μM. The final concentration of BUB1:BUB3, CDC20, and O-MAD2 (V193N) were 5 μM each. 50 μL of the solution was mixed with 15 μL of amylose beads (New England Biolabs). Samples were placed into Pierce micro-spin columns (Thermo Scientific) and incubated at 4 °C for 1 h. To separate the proteins bound to the amylose beads from the unbound proteins, the samples were centrifuged at 900×*g* for 2 min at 4 °C. The beads were washed three times with 200 μL of binding buffer. After the last washing step, 25 μL of elution buffer (binding buffer plus 10 mM maltose) was added to the column and centrifuged at 800×*g* for 2 min at 4 °C. The eluted proteins were mixed with 5× Laemmli sample buffer supplemented with β-mercaptoethanol and analyzed by SDS-PAGE and immunoblotting.

### Immunoprecipitation (IP) using mNeonGreen-Trap

HeLa-A12 cells integrated with the Tet-On expression cassette of either mNeonGreen, MAD1-mNG, or MAD1^ΔL^-mNG were induced to express the ectopic exogenous protein by 0.1 μg/mL doxycycline (for 2 days until being harvested) and arrested at mitosis using the thymidine–nocodazole synchronization protocol (see the next subsection on immunoblotting). Cells were harvested by mitotic shake-off, washed once by PBS, pelleted down by centrifugation at 200–500×*g* for 3 min, snap-frozen in liquid nitrogen, and stored at −80 °C before the IP experiment.

On the day of the IP experiment, cells were thawed on ice and lysed in the IP lysis buffer [75 mM HEPES-HCl (pH 7.5 at 4 °C), 150 mM KCl, 10% (by volume) glycerol, 1.5 mM MgCl_2_, 1.5 mM EGTA, and 1% (by mass) CHAPS] supplemented with 1 mM PMSF, the cOmplete EDTA-free Protease Inhibitor Cocktail, Phosphatase Inhibitor Cocktail IV (RPI), 1 mM Na_4_P_2_O_7_, 0.1 mM Na_3_VO_4_, 5 mM NaF, and 2 mM sodium β-glycerophosphate before usage. For 1 mg of wet cell pellet, 40 μL of 4 °C IP lysis buffer was added, yielding a total protein concentration of about 5.6 mg/mL (if cells were lysed completely). Resuspended cells were rotated for 30 min at 4 °C and then centrifuged at 18,000×*g* for 20 min at 4 °C. In total, 600 μL of supernatant was subsequently cleared to reduce non-specific binding by adding 50 μL of equilibrated control agarose beads (ChromoTek) and rotating for 45 min at 4 °C. The mixture was centrifuged at 2000×*g* for 5 min at 4 °C. In all, 580 μL of cleared supernatant was then mixed with 30 μL of equilibrated mNeonGreen-Trap Agarose (nta-20, ChromoTek) and rotated for 1 h at 4 °C. These beads were then pelleted down at 2000×*g* for 5 min at 4 °C to remove the supernatant. The beads were further washed four times (rotated for 5 min at 4 °C and then pelleted down at 2000×*g* for 5 min at 4 °C) using 1 mL of the IP wash buffer [75 mM HEPES-HCl (pH 7.5 at 4 °C), 150 mM KCl, 10% (by volume) glycerol, 1.5 mM MgCl_2_, and 1.5 mM EGTA] each time. The beads were transferred to a fresh tube before the last wash to avoid the non-specific binding of proteins to the wall of the tube. Finally, 2× Laemmli sample buffer supplemented with β-mercaptoethanol was added to the beads. Samples were boiled in a boiling water bath for 10 min and analyzed by SDS-PAGE and immunoblotting.

### Immunoblotting

To acquire unsynchronized HeLa-A12 cells, asynchronous cells were either scrapped or trypsinized off the surface of dishes. To acquire mitotic HeLa-A12 cells, cells were first synchronized in G1/S with 2.5 mM thymidine and then arrested in mitosis with 330 nM of nocodazole for 16 h. This procedure is referred to as the thymidine–nocodazole synchronization protocol.

Harvested cells were then washed once by PBS, pelleted down, and chilled on ice. Lysis was performed by directly mixing with 2× Laemmli sample buffer supplemented by β-mercaptoethanol at a ratio of 1 μL per 0.1 mg of cell pellets. Lysates were boiled immediately afterward for 10 min in a water bath and then chilled on ice. In all, 8 μL of supernatant was loaded onto each lane of a 15-well, 0.75-mm SDS-PAGE mini gel.

Primary antibodies (and their working dilution factors by volume) used included anti-BUBR1 (Bethyl Laboratories A300-995A-M, 1:1000), anti-BUB1 (Abcam ab9000), anti-CDC20 (Santa Cruz Biotechnology sc-5296 for Fig. 2F and sc-13162, 1:200 for others), anti-MAD2 (Bethyl Laboratories A300-301A-M, 1:330), anti-GAPDH (Proteintech 60004-1-Ig, 1:5000), anti-MAD1 (GeneTex GTX109519, 1:2000 for Supplementary Fig. 3E and PLA0092, 1:1000 for others), anti-mNeonGreen (Cell Signaling Technology 53061S, 1:100), and anti-BUB3 (Sigma-Aldrich B7811, 1:500).

### Amino acid sequences used for multiple sequence alignment and coiled-coil prediction

The following UniProt accession codes were used to retrieve Mad1 amino acid sequences for multiple sequence alignments or prediction of coiled-coil domains: human: Q9Y6D9-1, mouse: Q9WTX8-1, zebrafish: D9IWE2, African clawed frog: Q6GPD1, budding yeast: P40957, fission yeast: P87169.

### Statistics and reproducibility

In Fig. 1F, ordinary one-way ANOVA was used to compare the mean lifetime of the donor–acceptor cases with the donor-only case. Left: *F* = 14.84, right: *F* = 21.81, *P* < 0.0001 in both cases. The *P* values displayed in the Fig. were obtained using Dunnett’s test in GraphPad Prism.

### Reporting summary

Further information on research design is available in the Nature Portfolio Reporting Summary linked to this article.

## Supplementary information

Supplementary Information

Reporting Summary

## Data Availability

The fluorescence microscopy datasets generated for and/or analyzed during the current study are freely available from the corresponding author upon request. Source data are provided with this paper.

## Source data

Source Data

## Code availability

FLIM data analysis codes can be accessed at https://github.com/CreLox/FluorescenceLifetime.
