# Supplementary material for: The structural flexibility of MAD1 facilitates the assembly of the Mitotic Checkpoint Complex
Source: Nat Commun. 2023 Mar 18;14:1529. doi: 10.1038/s41467-023-37235-z (PMC10024682; doi:10.1038/s41467-023-37235-z)
Supplement: Supplementary file 1 — Supplementary Information [file 41467_2023_37235_MOESM1_ESM.pdf]

Supplementary Information

The Structural Flexibility of MAD1 Facilitates the Assembly of the Mitotic Checkpoint Complex

Chu Chen[1, 2], Valentina Piano[3, 4], Amal Alex[3], Simon J. Y. Han[5, 6], Pim J Huis In ’t Veld[3], Babhrubahan Roy[5], Daniel Fergle[5], Andrea Musacchio[3, 7], Ajit P. Joglekar[1, 5]

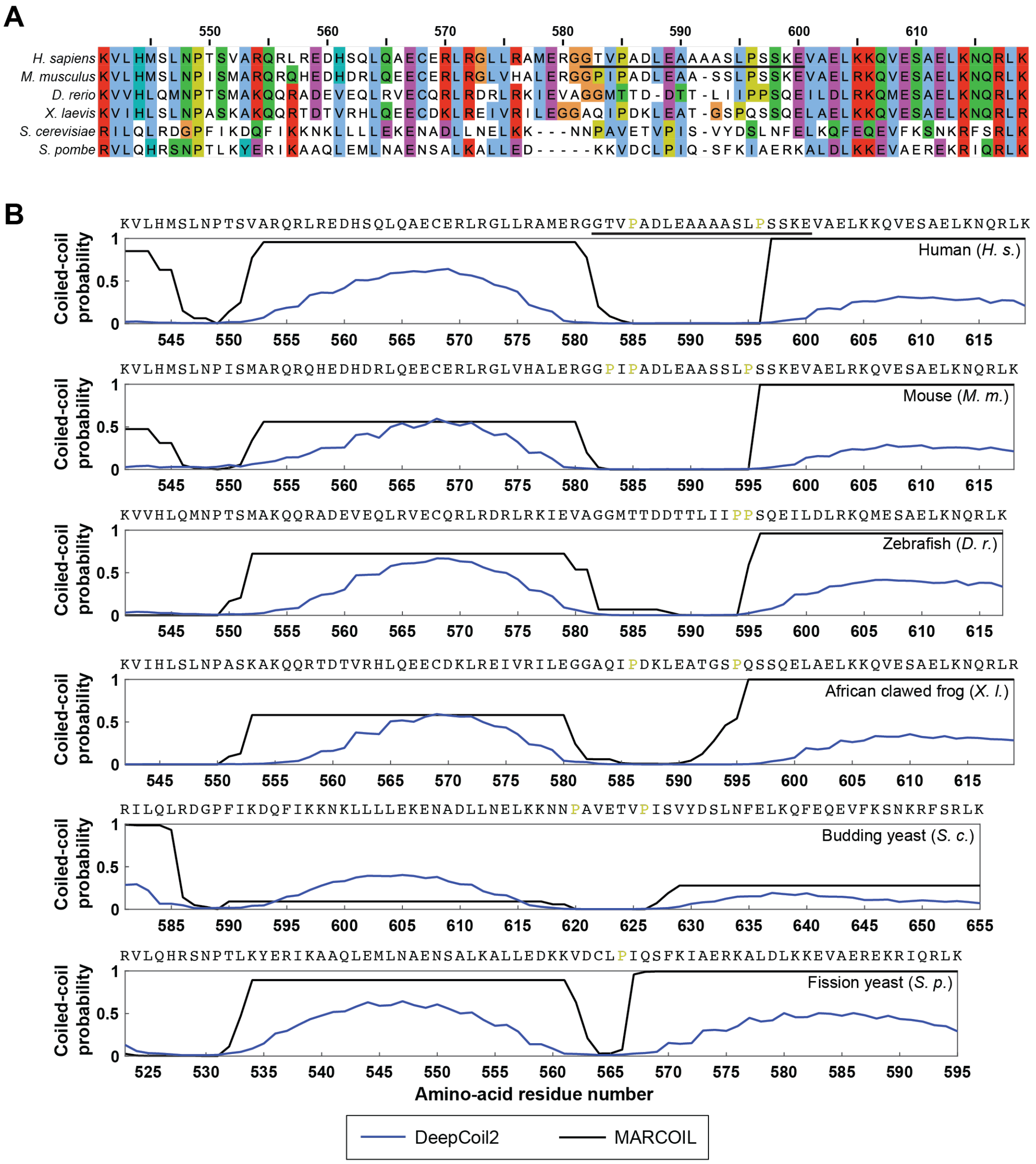

Supplementary Fig. 1. The secondary structure of the hinge of MAD1 is conserved.

(A) The primary sequence of MAD1's hinge is not conserved (Clustal X coloring scheme). Jalview was used in the multiple sequence alignment (using the MSAprobs alignment tool with default settings) and visualization. The amino-acid numbering at the top is for human MAD1. **(B)** Coiled-coil predictions using two algorithms (blue curves: raw predicted probabilities by DeepCoil2; black curves: MARCOIL) on the region spanning from MAD1's MIM (which is also not a coiled-coil [1]) to MAD1's consensus RLK motif from *Homo sapiens* (human), *Mus musculus* (mouse), *Danio rerio* (zebrafish), *Xenopus laevis* (African clawed frog), *Saccharomyces cerevisiae* (budding yeast), and *Schizosaccharomyces pombe* (fission yeast). The primary sequences of full-length MAD1 proteins were supplied as the input, but only probability predictions for the region spanning from the MIM to the RLK motif are shown. In both **(A)** and **(B)**, the segment encompassing residues 582–600 of human MAD1 is underlined. See Methods for the accession numbers used to retrieve amino acid sequences.

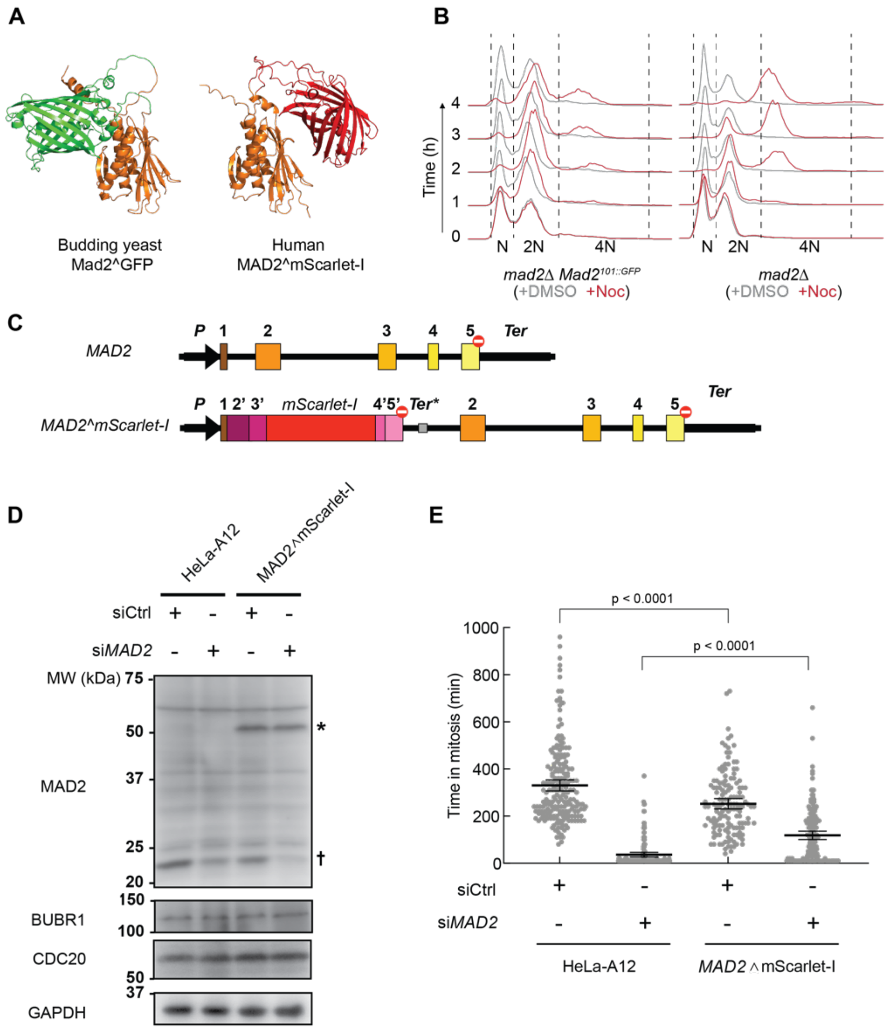

Supplementary Fig. 2. Internally tagged MAD2 is functional in budding yeast and human cells.
(A) A representative model of *Saccharomyces cerevisiae* Mad2∧GFP (left) and human MAD2∧mScarlet-I (right; fluorescent protein inserted within the β5-αC loop in both cases) predicted by the ColabFold advanced algorithm. **(B)** Effects of Nocodazole treatment on cellular DNA content in the indicated budding yeast strains. A population of asynchronously growing yeast cells contains a mixture of G1 (N) and G2/M (2N) cells. The 4N population corresponds to cells that slipped out of the mitotic block to reenter the cell cycle. Representative results from two experiments shown. **(C)** Diagram of the endogenous MAD2 allele and the genome-edited MAD2∧mScarlet-I allele. Boxes 1–5 represent the exons. Boxes 2’–5’ encode the same peptides as boxes 2–5 respectively, with the introduction of silence mutations that make MAD2∧mScarlet-I resistant to siMAD2. The black arrow represents the promoter and the 5’-UTR (P). The black bar represents the 3’-UTR and the polyadenylation signal (Ter). The gray bar represents the polyadenylation signal of rabbit β-globin (Ter*). The red stop signs represent stop codons. **(D)** Immunoblotting showed that MAD2∧mScarlet-I (labeled by an asterisk, expected molecular weight ~ 51.0 kDa) was correctly expressed in the heterozygous MAD2∧mScarlet-I HeLa-A12 cell line and was resistant against siMAD2. As a comparison, wild-type MAD2 (labeled by a cruciform with a molecular weight of 23.5kDa) was effectively knocked down by siMAD2. **(E)** Unsynchronized cells were treated with respective siRNAs for one day, treated with 50 nM nocodazole. Each gray dot represents a cell. The total number of cells in each group is N = 199, 143, 143, and 158 respectively. Mean values ±95% confidence intervals are overlaid. Results are representative of

two independent experiments. Unpaired two-sided t-tests with Welch's correction were performed in Prism. Source data are provided as a Source Data file.

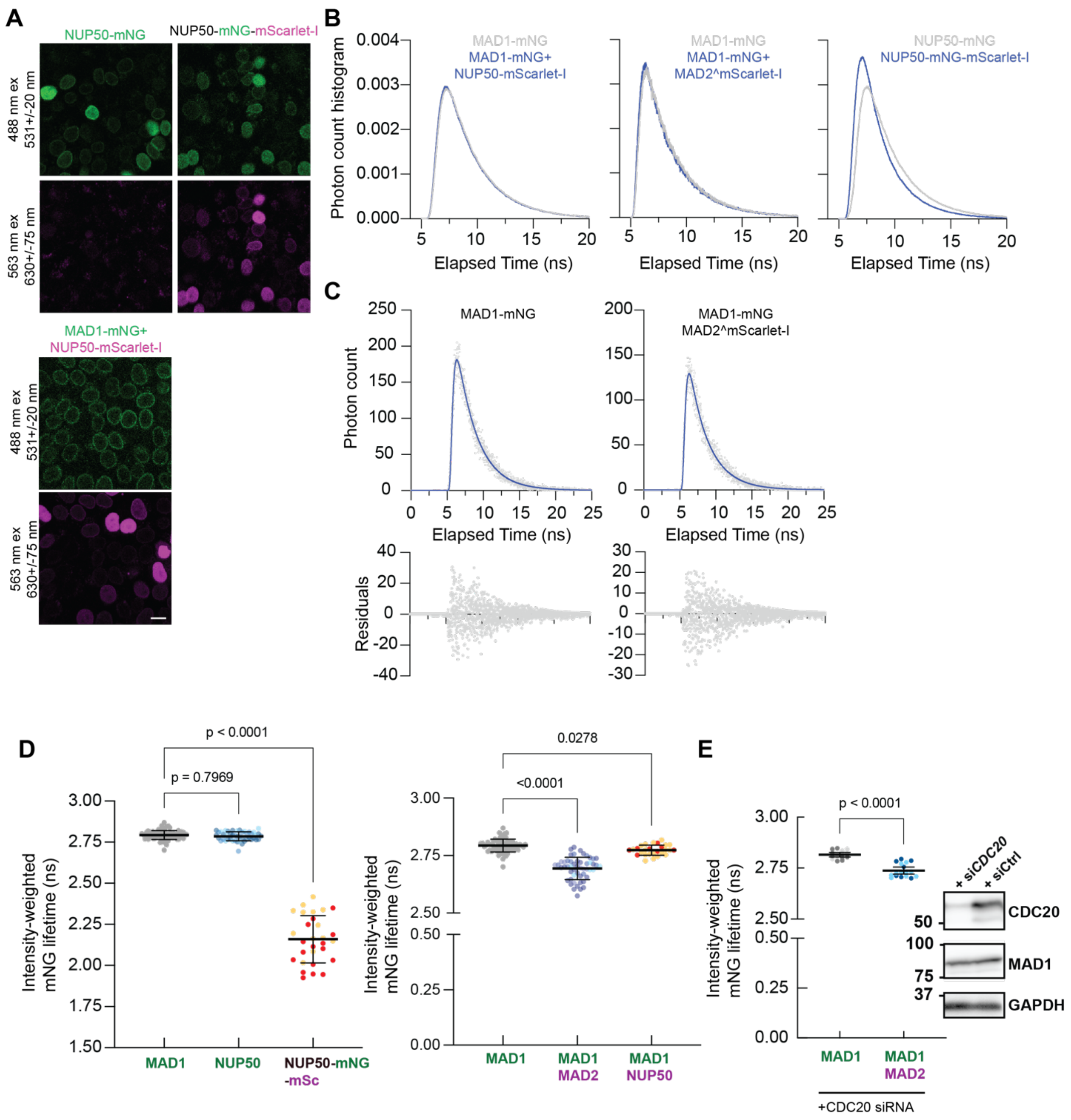

Supplementary Fig. 3. Validation of the FLIM data acquisition and analysis protocol.
(A) Representative images display the subcellular localizations of the indicated proteins (scale bar ~ 10 µm). **(B)** Top: averaged photon count histograms for either the donor alone (gray curve) or the donor in the presence of the indicated acceptor (blue curve; *N* = 17 from one trial out of three). **(C)** Representative fluorescence decay data (gray circles) for either MAD1-mNG alone (left) or MAD1-mNG in the presence of MAD2∧mScarlet-I (right). The blue curve displays the obtained fit. Corresponding residuals displayed in the bottom graphs. **(D)** Left: Excited-state lifetimes for MAD1-mNG, NUP50-mNG, and NUP50-mNG-mScarlet-I (*N* = 70, 46, and 29 pooled from three independent experiments). Ordinary one-way ANOVA ($F = 988$, $p < 0.0001$) was used to compare the mean lifetime values. Right: Excited-state lifetimes for MAD1-mNG alone or MAD1-mNG in the presence of either MAD2∧mScarlet-I or NUP50-mScarlet-I (data for MAD1-mNG and MAD2∧mScarlet-I replotted from Figure 1, *N* = 27 pooled from three independent experiments). Ordinary one-way ANOVA ($F = 121$ (2, 146), $p < 0.0001$)

was used to compare the mean lifetime values. **(E)** Excited-state lifetimes for MAD1-mNG alone and MAD1-mNG in the presence of MAD2ΛmScarlet-I after *CDC20* knock-down (*N* = 15 and 17 pooled from 2 independent experiments; Welch's *t*-test were used to compare the lifetimes). Right: Immunoblots of unsynchronized HeLa-A12 cells were treated with si*CDC20* or a control siRNA for 2 d and probed for CDC20, MAD1, and GAPDH (loading control). Observations in D and E are color coded by trial number. Source data are provided as a Source Data file.

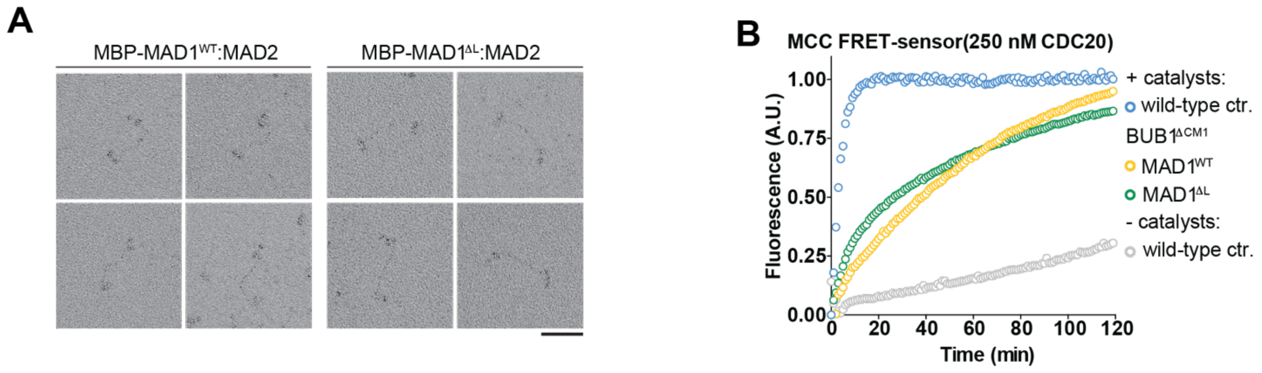

Supplementary Fig. 4. Characterization of the structure and *in vitro* activity of MAD1$^{\Delta L}$

(A) MBP-MAD1(wild-type or ΔL):MAD2 visualized by electron microscopy after glycerol spraying and low-angle platinum shadowing. Scale bars, 50 nm. Experiment performed twice. **(B)** MCC FRET-sensor-based assays show that when BUB1$^{\Delta CM1}$ is used instead of wild-type BUB1, MBP-MAD1:MAD2 (yellow) and MBP-MAD1$^{\Delta L}$:MAD2 (green) have a similar decreased activity in promoting MCC assembly. Curves report single measurements representative of at least three independent technical replicates. Source data are provided as a Source Data file.

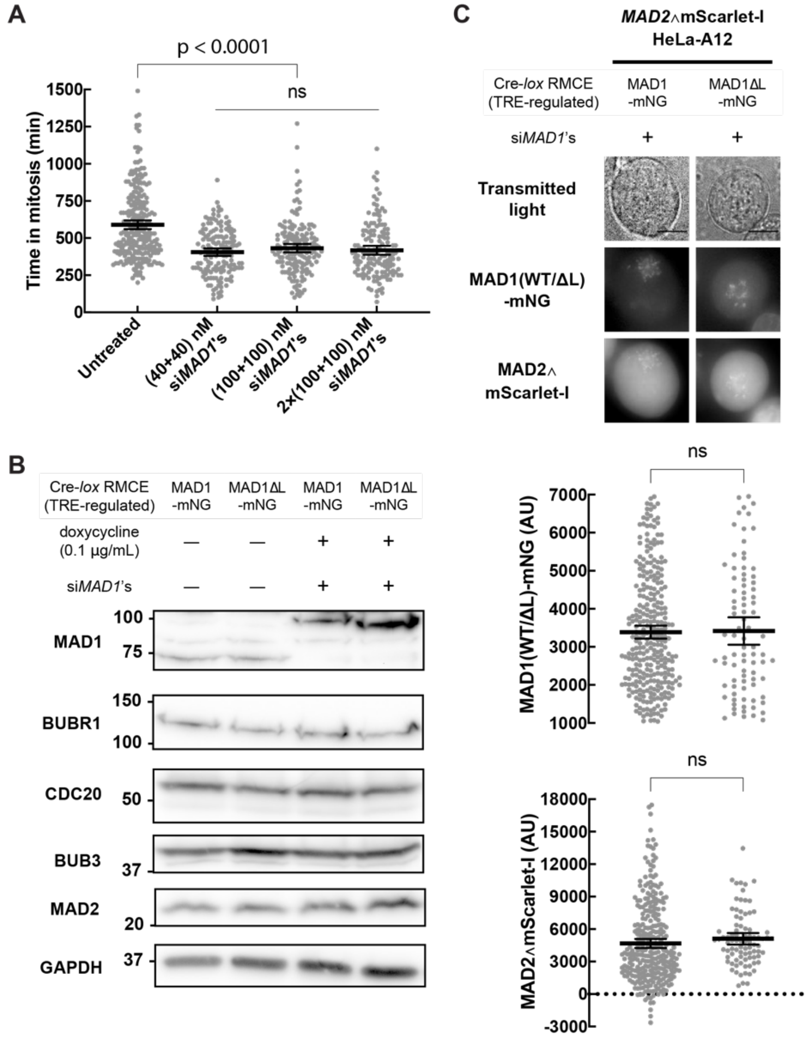

Supplementary Fig. 5. Deletion of the hinge does not affect the localization of the MAD1:MAD2 complex or the expression level of MCC constituents.

(A) MAD1 has a long half-life under normal conditions [2]. Like BUB1 [3-5], even a small pool of MAD1 (at less than 10% of its physiological concentration as quantified from Supplementary Fig. 5B) can maintain a considerable level of SAC signaling activity in nocodazole-treated cells. The conditions of siMAD1 treatment were (from left to right): untreated, 40nM each for two days (the standard condition used throughout this study), 100 nM each for two days, 100 nM each on day one and 100 nM each again on day two. Each dot represents a cell (N = 253, 150, 145, and 160 respectively). The mean value ± 95% confidence interval of each group is overlaid. Welch's ANOVA test [W(DFn, DFd) = 0.9885(2.000, 298.9), p = 0.3733] was performed for the three columns on the right. **(B)** siRNA treatment achieved over 90% knockdown of the endogenous MAD1, whereas the cellular abundance of either BUBR1, CDC20, or BUB3 was not affected. **(C)** The MAD2ΛmScarlet-I genome-edited HeLa-A12 treated with siMAD1's and rescued by MAD1WT/ΔL-mNG were imaged using wide-field fluorescence microscopy. Cells were arrested in mitosis using a thymidine–nocodazole synchronization protocol. Representative maximum z-projected images shown here share the same LUT. Scale bar, 10 μm. Due to variable induced expression levels of $MAD1^{WT/\Delta L}$-mNG in different cells, signaling kinetochores were filtered by the localization of $MAD1^{WT/\Delta L}$-mNG (with an arbitrary threshold of 1000–7000AU). Each gray dot represents a single signaling kinetochore (N = 298 and 85 respectively). The mean value ± 95% confidence intervals of each group is overlaid. Unpaired two-sided t-tests with Welch's correction was used to compare the mean intensities.

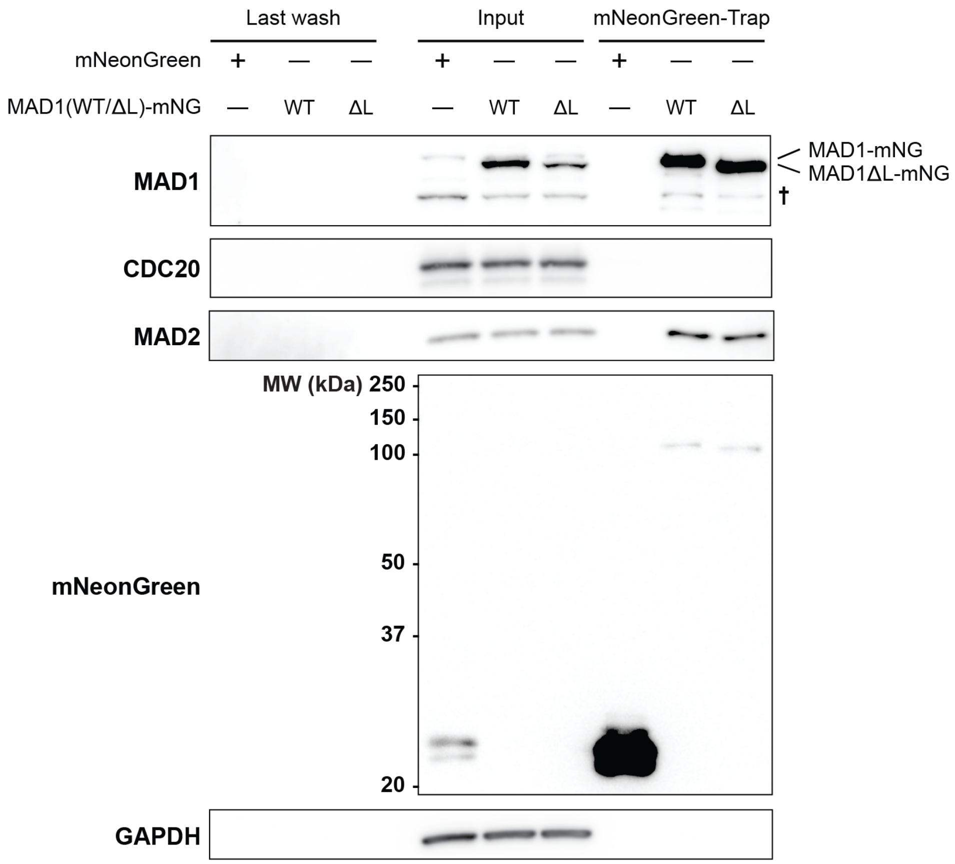

Supplementary Fig. 6. Immunoprecipitation of MAD1-mNG variants using mNeonGreen-Trap agarose. The cruciform symbol represents the endogenous MAD1 band. The expected molecular weights of the exogenous MAD1-mNG, $MAD1^{\Delta L}$-mNG, and mNG are 110.2 kDa, 108.4 kDa, and 26.9 kDa, respectively. The immunoblot against GAPDH served as the loading control. The immunoblots shown here are from the same immunoprecipitation experiment representative of two independent repeats.

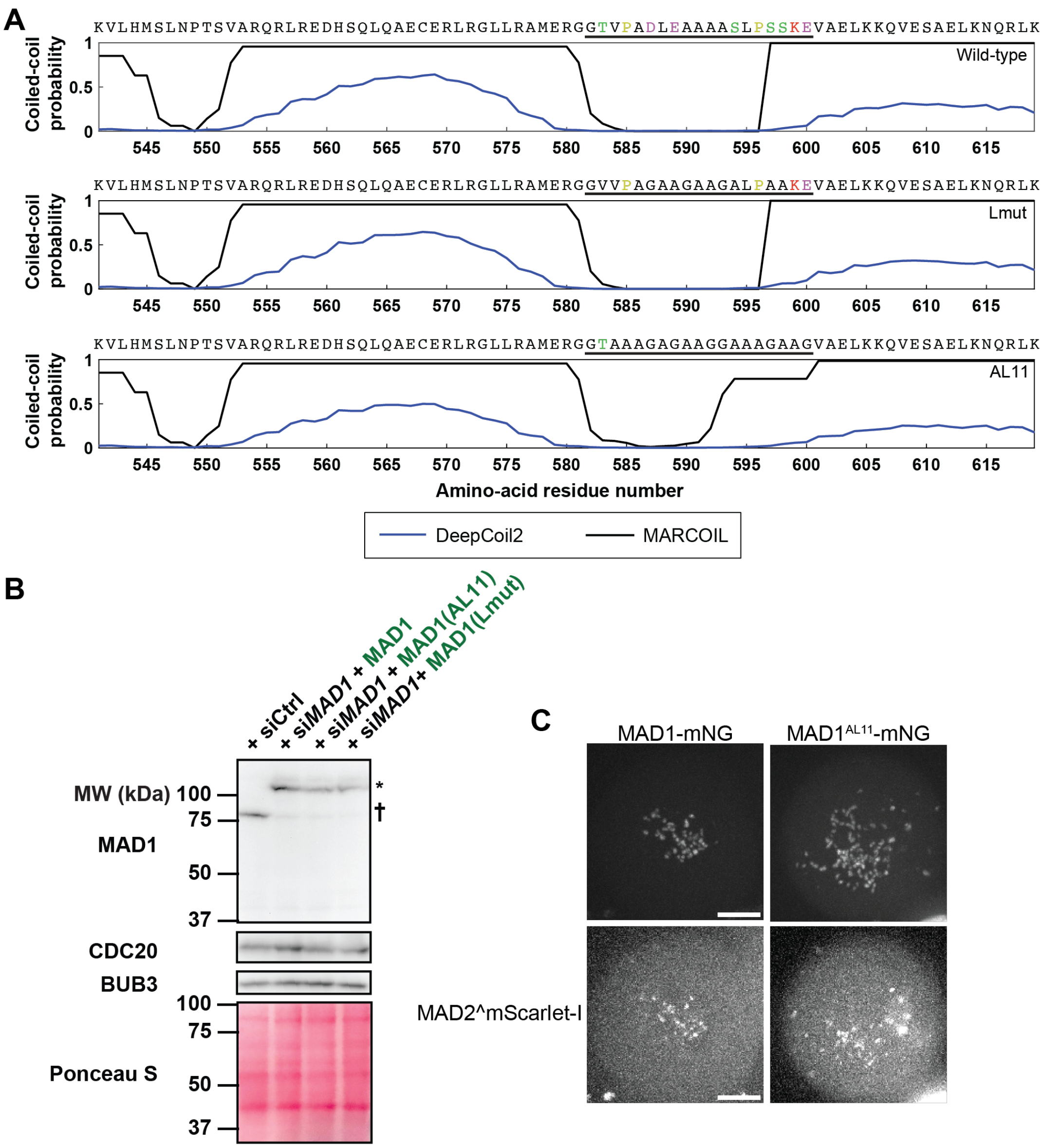

Supplementary Fig. 7. Coiled-coil predictions of MAD1 hinge replacement mutants reveal similar coiled-coil propensity profile as wild-type MAD1.

(A) The two algorithms and legends are the same as in Supplementary Fig. 1B. The top panel is reproduced from Supplementary Fig. 1B. Segments encompassing residues 582–600 are underlined. Serine/threonine residues are colored green. Proline residues are colored yellow. Negatively charged residues are colored purple. Lysine residues are colored red. (B) Immunoblot analysis of mitotic lysates of HeLa-A12 cells (first lane from the left) or HeLa-A12 cells expressing exogenous MAD1-mNG (second lane), $MAD1^{AL11}$-mNG (third lane), or $MAD1^{Lmut}$-mNG (fourth lane). Cells were treated with corresponding siRNAs and 0.1 μg/mL doxycycline for two days. Cells were synchronized by 2.5 mM thymidine overnight, released for 7 h, and treated with 330nM nocodazole for 4h before being harvested by the mitotic shake-off technique. The expected molecular weights of the exogenous MAD1-mNG, $MAD1^{AL11}$-mNG, and $MAD1^{Lmut}$-mNG are 110.2 kDa, 109.7 kDa, and 110.0 kDa, respectively. Ponceau S staining (bottom panel) of the MAD1 blot serves as a control for sample loading and membrane transfer (experiment was performed once). (C) Representative fluorescence micrographs displaying the localization of the indicated proteins (experiment repeated twice, scale bar = 5 μm).

SUPPLEMENTARY FIGURE 2

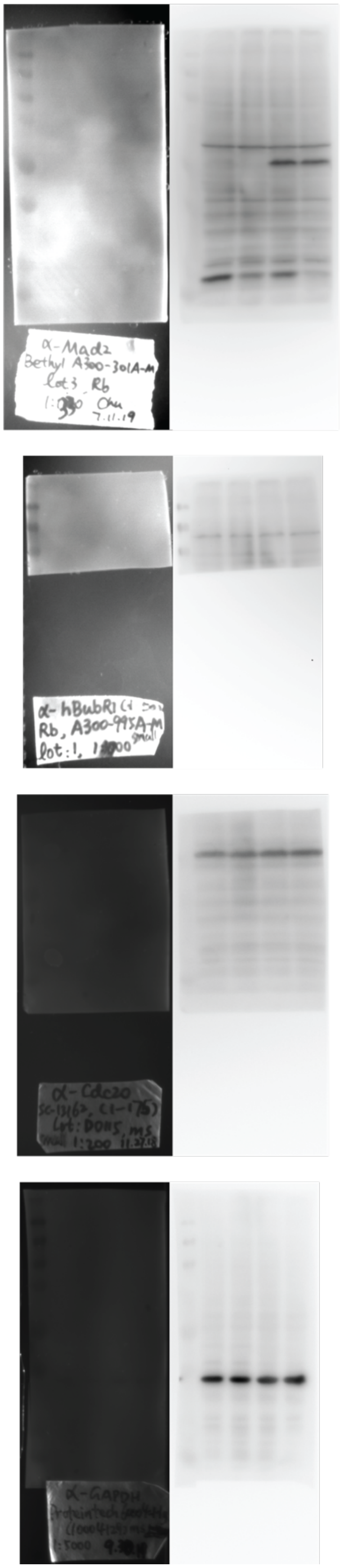

SUPPLEMENTARY FIGURE 3

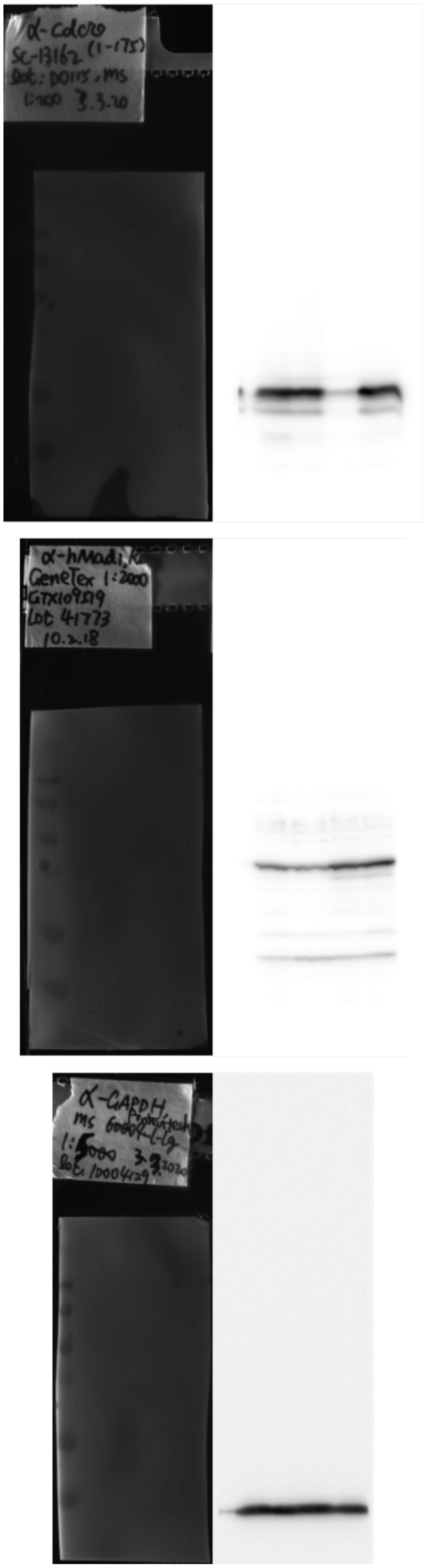

SUPPLEMENTARY FIGURE 5B

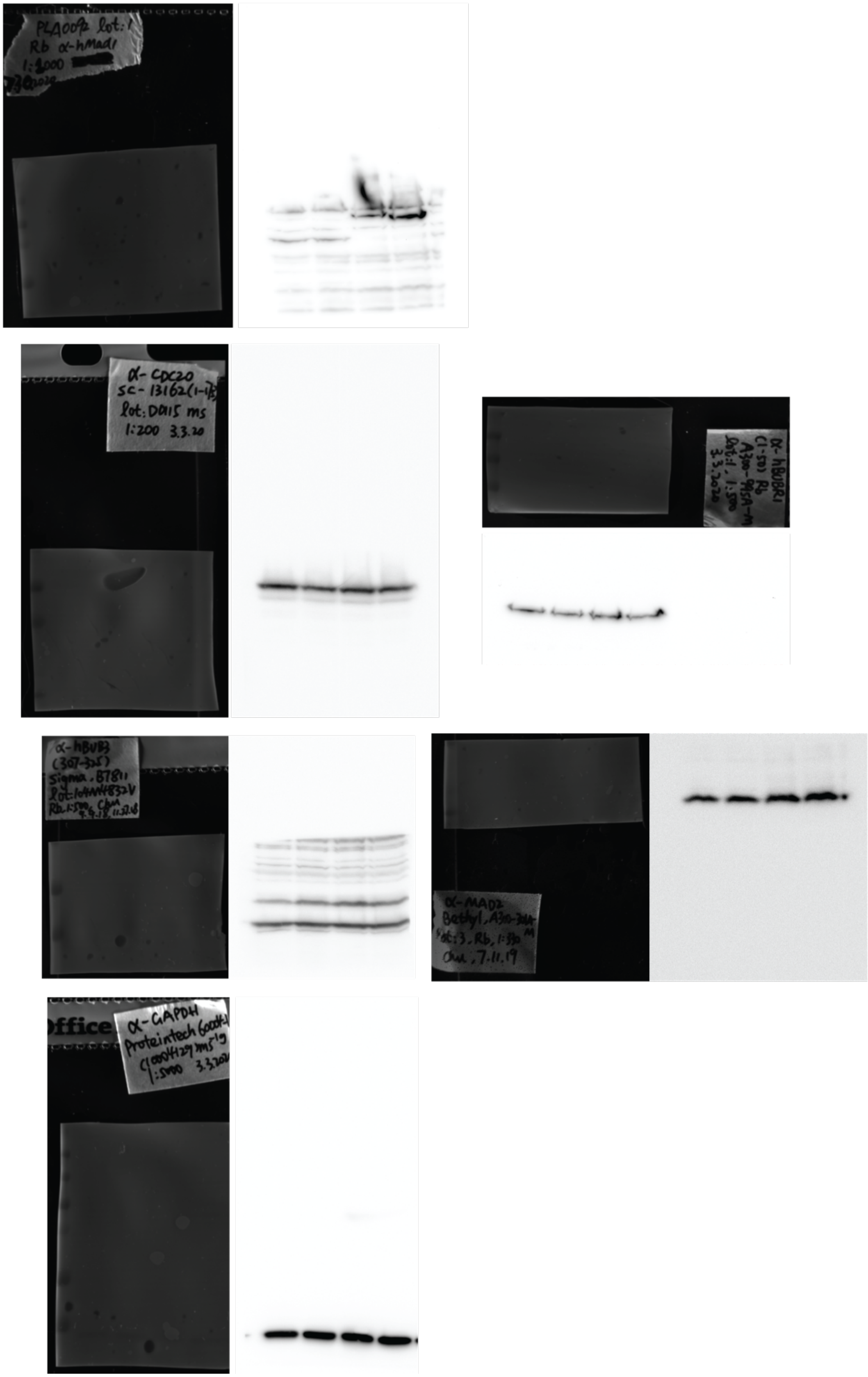

SUPPLEMENTARY FIGURE 6

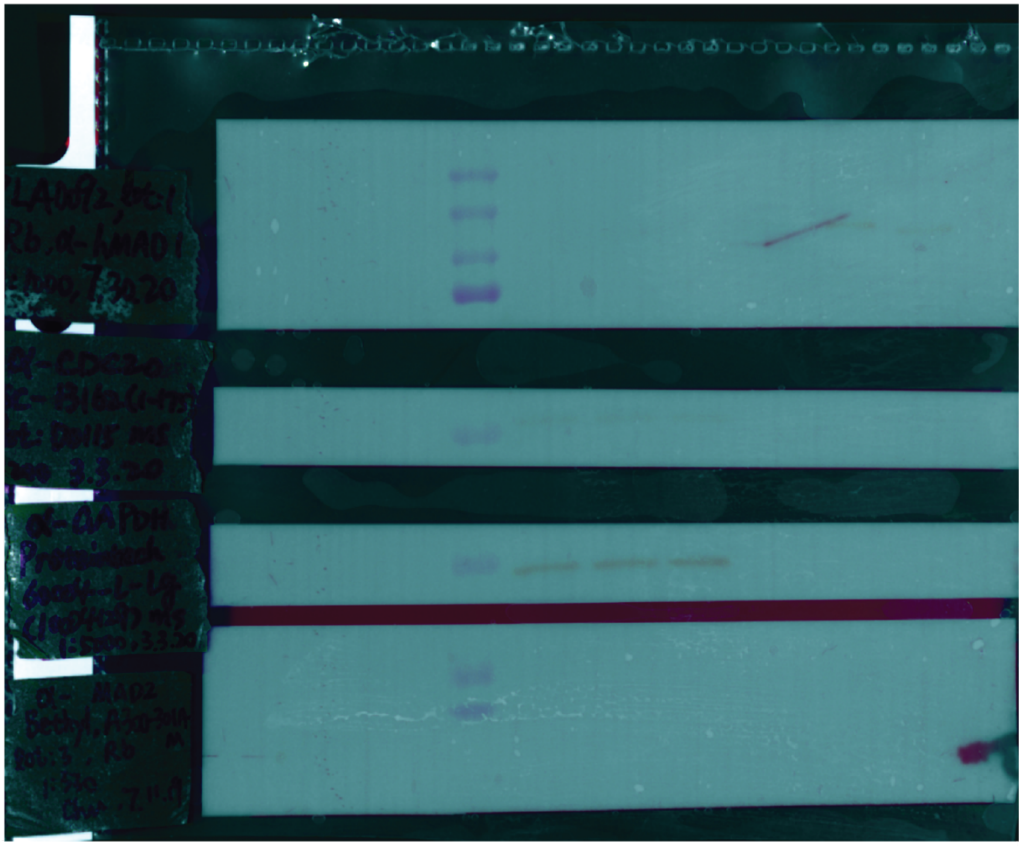

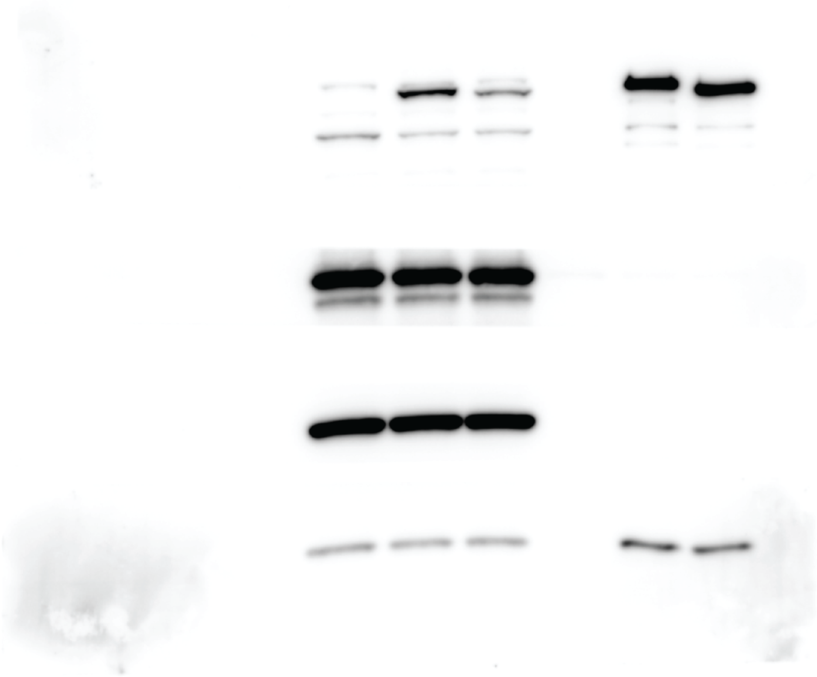

SUPPLEMENTARY FIGURE 6

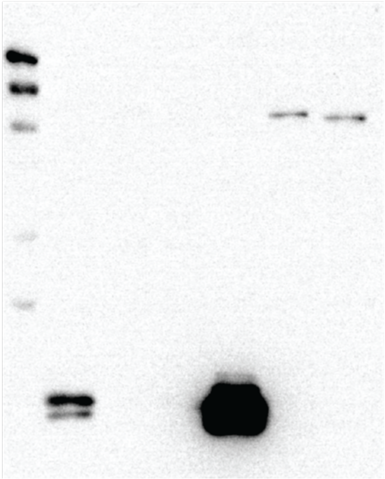

SUPPLEMENTARY FIGURE 7

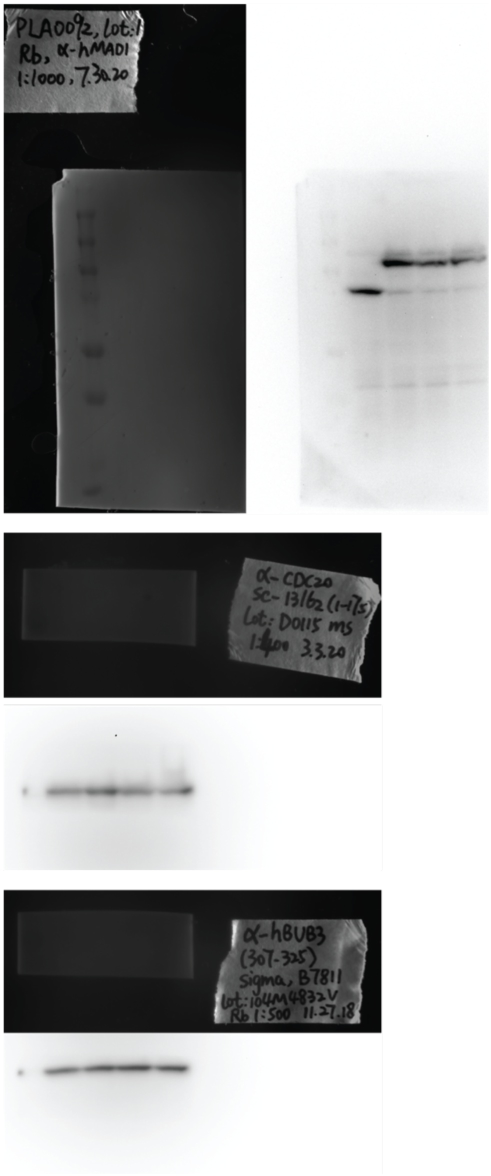

Supplementary References

1. Sironi, L. *et al.* Crystal structure of the tetrameric Mad1-Mad2 core complex: implications of a 'safety belt' binding mechanism for the spindle checkpoint. *EMBO J* **21**, 2496-2506 (2002).
2. Schweizer, N. *et al.* Spindle assembly checkpoint robustness requires Tpr-mediated regulation of Mad1/Mad2 proteostasis. *J Cell Biol* **203**, 883-893 (2013).
3. Raaijmakers, J.A. *et al.* BUB1 Is Essential for the Viability of Human Cells in which the Spindle Assembly Checkpoint Is Compromised. *Cell Rep* **22**, 1424-1438 (2018).
4. Rodriguez-Rodriguez, J.A. *et al.* Distinct Roles of RZZ and Bub1-KNL1 in Mitotic Checkpoint Signaling and Kinetochore Expansion. *Curr Biol* **28**, 3422-3429 e3425 (2018).
5. Zhang, G. *et al.* Efficient mitotic checkpoint signaling depends on integrated activities of Bub1 and the RZZ complex. *EMBO J* **38** (2019).
