# Supplementary material for: The structural flexibility of MAD1 facilitates the assembly of the Mitotic Checkpoint Complex
Source: Nat Commun. 2023 Mar 18;14:1529. doi: 10.1038/s41467-023-37235-z (PMC10024682; doi:10.1038/s41467-023-37235-z)
Supplement: Supplementary file 2 — Reporting Summary [file 41467_2023_37235_MOESM2_ESM.pdf]

nature portfolio

Corresponding author(s): Ajit P. Joglekar

Last updated by author(s): Feb 9, 2023

Reporting Summary

Nature Portfolio wishes to improve the reproducibility of the work that we publish. This form provides structure for consistency and transparency in reporting. For further information on Nature Portfolio policies, see our Editorial Policies and the Editorial Policy Checklist.

Statistics

For all statistical analyses, confirm that the following items are present in the figure legend, table legend, main text, or Methods section.

n/a	Confirmed	
☐	☒	The exact sample size (*n*) for each experimental group/condition, given as a discrete number and unit of measurement
☒	☐	A statement on whether measurements were taken from distinct samples or whether the same sample was measured repeatedly
☐	☒	The statistical test(s) used AND whether they are one- or two-sided<br>*Only common tests should be described solely by name; describe more complex techniques in the Methods section.*
☒	☐	A description of all covariates tested
☒	☐	A description of any assumptions or corrections, such as tests of normality and adjustment for multiple comparisons
☐	☒	A full description of the statistical parameters including central tendency (e.g. means) or other basic estimates (e.g. regression coefficient) AND variation (e.g. standard deviation) or associated estimates of uncertainty (e.g. confidence intervals)
☐	☒	For null hypothesis testing, the test statistic (e.g. *F*, *t*, *r*) with confidence intervals, effect sizes, degrees of freedom and *P* value noted<br>*Give P values as exact values whenever suitable.*
☒	☐	For Bayesian analysis, information on the choice of priors and Markov chain Monte Carlo settings
☒	☐	For hierarchical and complex designs, identification of the appropriate level for tests and full reporting of outcomes
☒	☐	Estimates of effect sizes (e.g. Cohen's *d*, Pearson's *r*), indicating how they were calculated

Our web collection on statistics for biologists contains articles on many of the points above.

Software and code

Policy information about availability of computer code

Data collection	VistaVision 4.2.153.0 used to collect all FLIM data
Data analysis	https://github.com/CreLox/IXNAnalysis (used in FIgure 3; the original publication, Chen, Chu et al. 2019, is cited)<br>https://github.com/CreLox/FluorescenceLifetime (used in Figure 1; this study)<br>Graphpad Prism 9<br>FlowJo_v10.7.1_CL<br>Matlab R2021

For manuscripts utilizing custom algorithms or software that are central to the research but not yet described in published literature, software must be made available to editors and reviewers. We strongly encourage code deposition in a community repository (e.g. GitHub). See the Nature Portfolio guidelines for submitting code & software for further information.

Data

Policy information about availability of data

All manuscripts must include a data availability statement. This statement should provide the following information, where applicable:

- Accession codes, unique identifiers, or web links for publicly available datasets
- A description of any restrictions on data availability
- For clinical datasets or third party data, please ensure that the statement adheres to our policy

We did not upload our microscopy data to a public repository because the total file size is too large. However, these data will be made freely available upon a reasonable request by the corresponding author.

Human research participants

Policy information about studies involving human research participants and Sex and Gender in Research.

Reporting on sex and gender	N/A
Population characteristics	N/A
Recruitment	N/A
Ethics oversight	N/A

Note that full information on the approval of the study protocol must also be provided in the manuscript.

Field-specific reporting

Please select the one below that is the best fit for your research. If you are not sure, read the appropriate sections before making your selection.

☒ Life sciences ☐ Behavioural & social sciences ☐ Ecological, evolutionary & environmental sciences

For a reference copy of the document with all sections, see nature.com/documents/nr-reporting-summary-flat.pdf

Life sciences study design

All studies must disclose on these points even when the disclosure is negative.

Sample size	Exact sample sizes have been specified in the figure legends. Sample sizes were not predetermined. Instead, we repeated each experiment at least two times. In microscopy experiments, we recorded images of a sufficiently large number of randomly selected fields of view and then analyzed the cells from these images. We ensured that this analysis produced reproducible results.
Data exclusions	These criteria were pre-established. In experiments associated with Figure 3, no data were excluded but the cells that were analyzed need to satisfy<br>1. Entered mitosis 20-1000 min after the start of imaging. This ensures a similarly short exposure to nocodazole during the interphase.<br>2. Had consistent (during the time course) and similar-to-physiological MAD1(WT/hinge-deleted)-mNG expression (see methods for details).<br>3. If two closely-located cells had similar expression levels of MAD1(WT/hinge-deleted)-mNG and entered and exited mitosis at about the same time, they were considered sister cells (progenies from the same parent cell) and only analyzed once.<br>4. Looked healthy with no obvious abnormalities in the interphase (for example, multinuclear, unusually large, having a large number of big vacuoles in the cytosol, bright foci in the nucleus in the green fluorescence channel, etc.) or during the mitosis (e.g., severe blebbing, undergoing apoptosis rather than anaphase onset, fusing with nearby cells, etc.).
Replication	At least two technical repeats (same cell lines -- not biological repeats) were included for each group. Data were pooled from the repeats. Each experimental repeat included a suitable positive or negative control group that tested the efficacy of the experimental treatment (e.g., RNAi) and/or the configuration of microscopy set-up. In rare instances, the results from the control group did not conform with the expectations. In these cases, we did not analyze the data.
Randomization	Cells were allocated into either control or experimental group based on their treatment history. During microscopy experiments, we selected the field of view randomly for each well or coverslip, taking into account only favorable cell density. Images were then recorded for these fields as per the protocol. During data anlaysis, all mitotic cells in the field of view were analyzed. These data were filtered if necessary based on pre-established criteria as documented in the Methods.
Blinding	Blinding is not possible because the same person set up the imaging experiments and performed data analysis. The images were collected, stored, and analyzed by well (on imaging plates), and the experimenter was aware of the type of cells and the treatment condition for each well on a plate.

Reporting for specific materials, systems and methods

We require information from authors about some types of materials, experimental systems and methods used in many studies. Here, indicate whether each material, system or method listed is relevant to your study. If you are not sure if a list item applies to your research, read the appropriate section before selecting a response.

Materials & experimental systems

n/a	Involved in the study
☐	☒ Antibodies
☐	☒ Eukaryotic cell lines
☒	☐ Palaeontology and archaeology
☒	☐ Animals and other organisms
☒	☐ Clinical data
☒	☐ Dual use research of concern

Methods

n/a	Involved in the study
☒	☐ ChIP-seq
☐	☒ Flow cytometry
☒	☐ MRI-based neuroimaging

Antibodies

Antibodies used	anti-BUBR1 (Bethyl Laboratories A300-995A-M, 1 : 1000), anti-BUB1 (Abcam ab9000), anti-CDC20 (Santa Cruz Biotechnology sc-5296 for Figure 2F and sc-13162, 1 : 200 for others), anti-MAD2 (Bethyl Laboratories A300-301A-M, 1 : 330), anti-GAPDH (Proteintech 60004-1-Ig, 1 : 5000), anti-MAD1 (GeneTex GTX109519, 1 : 2000 for Figure S3E and PLA0092, 1 : 1000 for others), anti-mNeonGreen (Cell Signaling Technology 53061S, 1 : 100), and anti-BUB3 (Sigma-Aldrich B7811, 1 : 500).
Validation	Antibody validation as provided on the manufacturer's website. Data are also provided in the manuscript.

Eukaryotic cell lines

Policy information about cell lines and Sex and Gender in Research

Cell line source(s)	Khandelia et al., 2011 (parent HeLa-A12); this study (transformed HeLa-A12 cell lines that express exogenous proteins, genome-edited MAD2^mScarlet-I and MAD1-mNG/MAD2^mScarlet-I HeLa-A12); the genome-edited MAD1-mNG HeLa-A12 was made by the Joglekar lab and described in Banerjee et al. 2022 MBoC (https://doi.org/10.1091/mbc.E22-03-0085)
Authentication	The parent HeLa-A12 cell line was generated in Khandelia et al., 2011. We did not further authenticate it. Transformed HeLa-A12 cell lines generated in this study that express exogenous proteins were confirmed by genotyping at the locus of Cre-lox RMCE integration. Genome-edited (via CRISPR-Cas9) HeLa-A12 were confirmed by genotyping and sequencing (near PAMs).
Mycoplasma contamination	Cells were regularly tested and we confirmed no contamination in experiments of this study according to PCR-based tests.
Commonly misidentified lines (See ICLAC register)	No commonly mis-identified cell lines were used in this study.

Flow Cytometry

Plots

Confirm that:

☒ The axis labels state the marker and fluorochrome used (e.g. CD4-FITC).

☒ The axis scales are clearly visible. Include numbers along axes only for bottom left plot of group (a 'group' is an analysis of identical markers).

☒ All plots are contour plots with outliers or pseudocolor plots.

☒ A numerical value for number of cells or percentage (with statistics) is provided.

Methodology

Sample preparation	Flow cytometry was used only to quantify the DNA content in a population of yeast cells. Yeast strains were grown to mid logarithmic phase and then treated with nocodazole to depolymerize microtubules (final concentration 15µg/ml). Sample aliquots containing approximately 0.1 OD600 were collected at 0, 1-, 2-, 3- and 4-hour time points after nocodazole addition. The cell samples were fixed by 70% ethanol and stored at 4 C overnight. Next day, the samples were washed and treated with bovine pancreatic RNase (Millipore Sigma, final concentration 170ng/ml) at 37 C for 24 h in RNase buffer (10mM Tris pH8.0, 15mM NaCl). Next day, the samples were washed again and resuspended in 1x phosphate buffered saline (PBS, pH 7.4) and stored at 4 C. The samples were treated with propidium iodide (Millipore Sigma, final concentration 5mg/ml in PBS) for 2h at RT on the day of the experiment.

Instrument	LSR Fortessa (BD Biosciences)
Software	FlowJo_v10.7.1_CL
Cell population abundance	Data was collected for ~ 10,000 events in each experiment. Gating was used to remove debris and non-singlet events.
Gating strategy	Single yeast cells were gated based on forward and side scatter, followed by forward scatter and Propidium iodide (PI) signal (610/20) to remove fluorescent debris. The PI signal from the entire cell population was plotted as a histogram.

☒ Tick this box to confirm that a figure exemplifying the gating strategy is provided in the Supplementary Information.
